# Poorly Conserved P15 Proteins of Cileviruses Retain Elements of Common Ancestry and Putative Functionality: A Theoretical Assessment on the Evolution of Cilevirus Genomes

**DOI:** 10.3389/fpls.2021.771983

**Published:** 2021-11-05

**Authors:** Pedro L. Ramos-González, Tirso Pons, Camila Chabi-Jesus, Gabriella Dias Arena, Juliana Freitas-Astua

**Affiliations:** ^1^Laboratório de Biologia Molecular Aplicada, Instituto Biológico de São Paulo, São Paulo, Brazil; ^2^National Centre for Biotechnology (CNB-CSIC), Madrid, Spain; ^3^Escola Superior de Agricultura Luiz de Queiroz (ESALQ), Universidade de São Paulo, Piracicaba, Brazil; ^4^Embrapa Mandioca e Fruticultura, Cruz das Almas, Brazil

**Keywords:** *Kitaviridae*, orphan ORF, structure-based phylogenetic analysis, synonymous codon usage bias, horizontal gene transfer, *Dichorhavirus*, miniproteins, small ORF

## Abstract

The genus *Cilevirus* groups enveloped single-stranded (+) RNA virus members of the family *Kitaviridae*, order *Martellivirales*. Proteins P15, scarcely conserved polypeptides encoded by cileviruses, have no apparent homologs in public databases. Accordingly, the open reading frames (ORFs) *p15*, located at the 5′-end of the viral RNA2 molecules, are considered orphan genes (ORFans). In this study, we have delved into ORFs *p15* and the relatively poorly understood biochemical properties of the proteins P15 to posit their importance for viruses across the genus and theorize on their origin. We detected that the ORFs *p15* are under purifying selection and that, in some viral strains, the use of synonymous codons is biased, which might be a sign of adaptation to their plant hosts. Despite the high amino acid sequence divergence, proteins P15 show the conserved motif [FY]-L-x(3)-[FL]-H-x-x-[LIV]-S-C-x-C-x(2)-C-x-G-x-C, which occurs exclusively in members of this protein family. Proteins P15 also show a common predicted 3D structure that resembles the helical scaffold of the protein ORF49 encoded by radinoviruses and the phosphoprotein C-terminal domain of mononegavirids. Based on the 3D structural similarities of P15, we suggest elements of common ancestry, conserved functionality, and relevant amino acid residues. We conclude by postulating a plausible evolutionary trajectory of ORFans *p15* and the 5′-end of the RNA2 of cileviruses considering both protein fold superpositions and comparative genomic analyses with the closest kitaviruses, negeviruses, nege/kita-like viruses, and unrelated viruses that share the ecological niches of cileviruses.

## Introduction

Orphan genes (ORFans) code for proteins with unrecognized homologs in any other species (Fischer and Eisenberg, 1999; Arendsee et al., 2014). They are present in a variety of organisms, including prokaryotes, eukaryotes, and viruses, reaching up to one-third of the genome of some microorganisms and 50% to two-thirds in mimiviruses (Fischer and Eisenberg, 1999; Kaur Saini and Fischer, 2007; Yin and Fischer, 2008; Tautz and Domazet-Lošo, 2011; Colson et al., 2017). The quick origin of ORFans may provide adaptive mechanisms conferring rapid fitness to changing environments (Entwistle et al., 2019). Most of the known proteins encoded by ORFans interact with conserved proteins like transcription factors or receptors, acting as toxins, or as modulators of metabolic or regulatory networks (Singh and Wurtele, 2020).

*Kitaviridae*, order *Martellivirales*, is a family of heterogeneous plant-infecting viruses displaying two, three, or four segments of single-stranded positive-sense RNA molecules as genomes, which have been assigned into the genera *Cilevirus*, *Higrevirus*, and *Blunervirus*, respectively (Quito-Avila et al., 2021). Taken together, kitavirids have been detected in a relatively narrow range of natural hosts, where they normally produce local infections mainly characterized by chlorotic and/or necrotic lesions that in some cases resemble the outcome of a hypersensitive-like response (Arena et al., 2016, 2020). However, regardless of their failure to consummate the systemic movement through the plants, kitavirids pose serious threats to major crops such as citrus (Ramos-González et al., 2018). Likely most kitaviruses are transmitted by mites, but an effective transmission by *Brevipalpus* mites has been only confirmed for cileviruses (Kitajima and Alberti, 2014; Quito-Avila et al., 2021).

**FIGURE 1:**
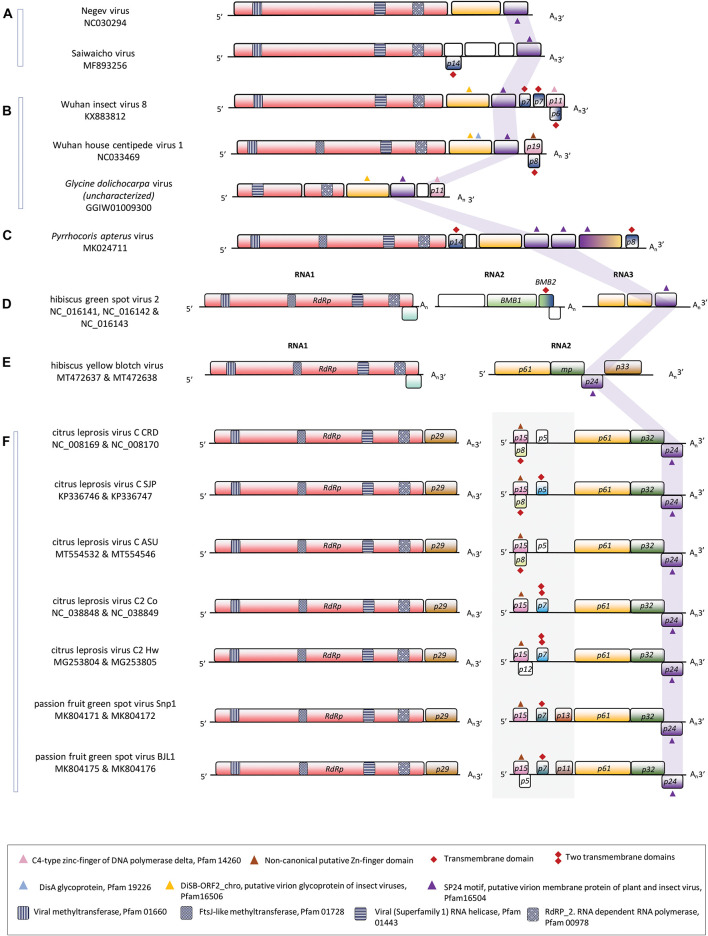
Linear genomic maps of some members of the family *Kitaviridae* and some of their closest phylogenetically arthropod-infecting viruses. **(A)** Two members of the group Negevirus, **(B)** two members of the group centivirus, **(C)** unclassified kita/nege-like virus, **(D)** the sole member of the genus *Higrevirus*, **(E)** a cile-like virus, and **(F)** members of the genus *Cilevirus*. Open reading frames are represented by boxes and the fill colors indicate a conserved functional or structural relationship between them. White boxes indicate unknown features. The solid gray background box in **(F)** highlights the 5′-end of the RNA2 genomic segment of cileviruses. The purple ribbon points out the position of the ORFs containing the SP-24 motif (structural protein of 24 kDa, Pfam code PF16504). Small geometric symbols show the presence of relevant sequences as described in the legend box.

With several strains and isolates belonging to three species already described at the biological and molecular level, *Cilevirus* is the best-studied genus of kitavirids (Freitas-Astúa et al., 2018; Quito-Avila et al., 2021). Virions of citrus leprosis virus C (CiLV-C), citrus leprosis virus C2 (CiLV-C2), and passion fruit green spot virus (PfGSV) are enveloped, short bacilliform particles that encapsidate two poly-adenylated RNA molecules (Locali-Fabris et al., 2006; Roy et al., 2013; Kitajima and Alberti, 2014; Ramos-González et al., 2020). The canonical genome of cileviruses contains six open reading frames (ORF) distributed in two RNA segments. ORFs *RdRp* (RNA dependent-RNA polymerase) and *p29* (putative coat protein) are in RNA 1 (≈9.0 kb); whilst the ORFs *p32* (movement protein), *p61*, *p24*, and *p15* are contained in the RNA2 (≈5.0 kb) (Figure 1). RNA2 in certain cileviruses also includes some accessory ORFs, for instance, *p7* in CiLV-C2, and *p11*-*13* in PfGSV. ORFs *p61* and *p24* encode proteins that are likely involved in the virion structure (Solovyev and Morozov, 2017). P24 is a transmembrane (TM) protein that is also probably an integral component of the virus factory-like membranes, whereas P61 is a putative glycoprotein that in the case of CiLV-C acts as a viral effector in plants (Kuchibhatla et al., 2014; Arena et al., 2016, 2020; Leastro et al., 2018; Vinokurov and Koloniuk, 2019). ORF *p61* is taxonomically restricted to cileviruses, whereas, besides in the kitavirids, *p24* is also present in an increasingly discovered number of arthropod-infecting viruses including mosquito-specific viruses of the proposed group Negevirus (clades Nelorpivirus and Sandewavirus) (Vasilakis et al., 2013; Kuchibhatla et al., 2014; Kondo et al., 2020). P24 displays the highly conserved motif SP24 (TM-SP24, structure protein of 24 kDa, Pfam code PF16504). The glycoproteins P61 of cileviruses lack the motifs DiSA (Pfam code PF19226) and DiSB-ORF2_chro B (Pfam code PF16506) detected in the glycoproteins encoded by their taxonomically related arthropod-infecting viruses (Kuchibhatla et al., 2014). ORF *p15*, only present in cileviruses, encodes the smallest annotated polypeptide across the genomes of these viruses (Ramos-González et al., 2020).

In this study, based on an integrator process comprising genomics, population genetics, usage of synonymous codons, and tridimensional (3D) structures, we elaborated a hypothesis about the conserved putative functionality of the P15 proteins across the genus. Following a chain of logical thinking, we theorize on the origin and evolution of the ORF *p15* considering the genomic context in the 5′-end of the RNA2 in cileviruses. A meaningful part of the study has been founded on the comparisons of the protein folds using the principles of structure-based phylogenetic analyses.

## The Open Reading Frames *p15* of Cileviruses Are Under Purifying Selection and Encode Poorly Conserved Proteins

Citrus leprosis virus C is the best-characterized cilevirus at both molecular and epidemiological levels. Its population is subdivided into three clades (Ramos-González et al., 2016; Chabi-Jesus et al., 2021). Average identity values of genomic nucleotide sequences between CiLV-C isolates belonging to different lineages range from 85 to 89%. Contrastingly, a highly conserved segment of approximately 1.5 kb, showing almost 100% of identity, is present at the 5′-end of their RNA2 molecules and comprises the ORF *p15* (393 nts) (Figure 1).

**FIGURE 2:**
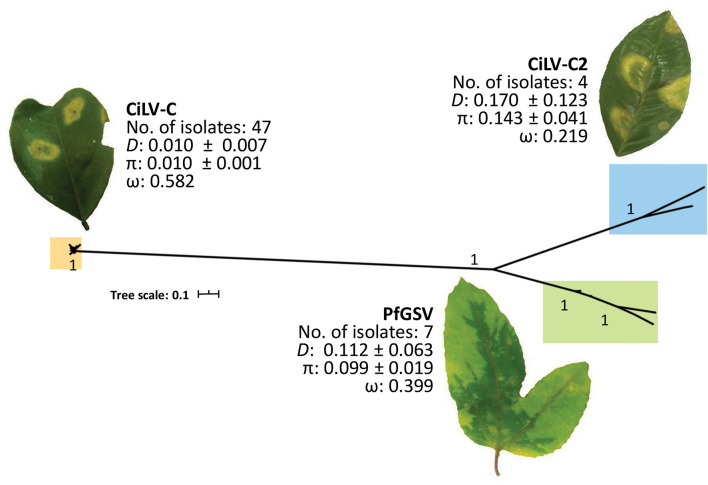
Phylogenetic analysis of P15 proteins and intra-species variability of ORF *p15* from cileviruses. Nucleotide sequence alignments were performed using the MAFFT algorithm (Katoh and Standley, 2013). The best-fit nucleotide substitution model was estimated using MEGA X v. 10.1.8 (Kumar et al., 2018). Model HKY + G (Hasegawa et al., 1985), with the lowest Bayesian information criterion (BIC), was used for the estimation of the phylogenetic relationships. The phylogenetic tree was inferred using the Maximum Likelihood method implemented in the Galaxy platform (Afgan et al., 2018). The reliability of the inferred evolutionary relationships was assessed by 1,000 bootstrapped replications. The tree was edited and visualized using Interactive Tree Of Life (iTOL) v4 (Letunic and Bork, 2019). ORF *p15* sequences from 47, 4, and 7 isolates of CiLV-C, CiLV-C2, and PfGSV, respectively, were retrieved from the GenBank database accessed on 17th March 2020 (Supplementary Table 1). Intraspecific genetic distance (*D*) and Non-synonymous (dN) and synonymous (dS) rates of substitutions (ω) were calculated in MEGA X, and nucleotide diversity (π) and the confidence intervals for π values were estimated using DnaSP software v6.12.03 (Rozas et al., 2017). Photo of the CiLV-C2-infected orange leaf is courtesy of Dr. Guillermo Adolfo Leon Martinez, AGROSAVIA, 2021.

**FIGURE 3:**
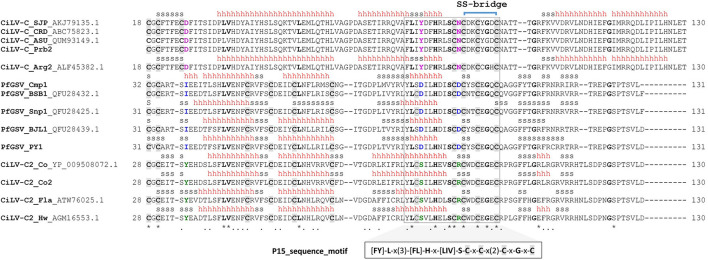
Consensus sequence obtained from a multiple sequence alignment (MSA) of the cysteine-rich domain of the P15 protein family. MSA was built using ClustalO (Sievers and Higgins, 2014). The alignment was manually curated by analyzing gaps, conserved amino acid positions, and the predicted secondary structure. The non-aligning segments at the N-terminus of P15 proteins, presumably representing non-core regions, were removed. Conserved cysteine residues are highlighted by gray background and boldface letters. Secondary structure elements, as predicted by I-TASSER, are depicted on top. Helix and β-strand conformations are indicated by h and s, respectively. A conserved sequential motif {[FY]-L-x(3)-[FL]-H-x-[LIV]-S-C-x-C-x(2)-C-x-G-x-C} is displayed below the sequence alignment. Strictly conserved residues are in boldface and putative functional residues specific from each group are in magenta, blue, and green colors. The putative disulfide bridge between the cysteine residues (positions 85 and 92 in CiLV-C) is represented by a blue bracket. Asterisk and point symbols beneath the alignment indicate strictly conserved residues and conservative substitutions, respectively.

Based on the analysis of 58 nucleotide sequences of *p15* (Supplementary Table 1) using MEGA v. 10.1.8 (Kumar et al., 2018) and DnaSP v. 6.12.03 (Rozas et al., 2017) software suites, we confirmed that CiLV-C *p15* variability is very low with an overall genetic distance (*D*) and nucleotide diversity (π) ≤ 0.01 (Figure 2). According to population analyses, this low variability may result from constant purges of the non-synonymous substitutions by purifying selection (ω < 1), and/or intra-species recombination processes, as already described (Ramos-González et al., 2016; Chabi-Jesus et al., 2021). P15 encoded by CiLV-C shows Cys residues that were earlier suggested as taking part in a putative Zn-finger structure (Ramos-González et al., 2016) (Figure 3). Transiently expressed P15 of CiLV-C of the strain CRD enters into the cell nucleus likely by passive diffusion and the formation of homodimers and heterodimers with P29 and the viral movement proteins can be detected in the cytosol of agroinfiltrated *Nicotiana benthamiana* plants (Leastro et al., 2018). When expressed from a viral vector, the infected *N. benthamiana* plants show stunted growth and enhanced necrosis in their younger leaves suggesting pathophysiological disorders caused by its ectopic expression. A possible role as an RNA silencing suppressor was also suggested for P15 of CiLV-C strain CRD (Leastro et al., 2020).

Analyses conducted in this work indicated that despite the low number of isolates available for CiLV-C2 and PfGSV (Supplementary Table 1), intra-species variability of their *p15* is almost 10-fold higher than among the CiLV-C isolates. Yet, as well as for CiLV-C, ORFs *p15* from CiLV-C2 and PfGSV show signatures of purifying selection (ω < 1). P15 proteins of these two viruses are phylogenetically closer to each other and more distant from that in CiLV-C (Figure 2).

Pairwise comparisons of the P15 amino acid (aa) sequences reveal very low identity values, approximately 14 or 20%, between the proteins from CiLV-C and PfGSV or CiLV-C2, respectively. Higher values, still moderate, approximately 55% aa sequence identity, are detected in the comparisons between P15 from CiLV-C2 and PfGSV (Ramos-González et al., 2016, 2020). Remarkably, the aa pairwise sequence identity values among P15 of CiLV-C and those from CiLV-C2 and PfGSV meaningfully deviate from those commonly observed within the cilevirus proteomes. The identity values between the P15 of PfGSV and CiLV-C are less than half of those shown by the next less conserved proteins among cileviruses, i.e., P29 and P61 (31–32%) (Ramos-González et al., 2020).

## Open Reading Frame *p15* Displays Synonymous Codon Usage Bias That Might Reflect Adaption to Host-Specific Codon Usage Patterns

Codon usage bias (CUB) refers to differences in the frequency of occurrence of synonymous codons in coding sequences (Behura and Severson, 2013). In viruses, CUB reflects changes in gene expression that occur as a consequence of, for instance, the interplay and co-evolution with their hosts, which may increase the viral fitness (Biswas et al., 2019; He et al., 2019; Khandia et al., 2019). Several indices, such as the Effective Number of codons (ENc), the Relative Synonymous Codon Usage (RSCU), COdon Usage Similarity Index (COUSIN), and Relative Codon Deoptimization Index (RCDI) are used to quantify the CUB (Sharp and Li, 1987; Wright, 1990; Bourret et al., 2019). These indices enable the comparison of genes within a genome and across different genotypes and species revealing elements about their evolution.

**FIGURE 4:**
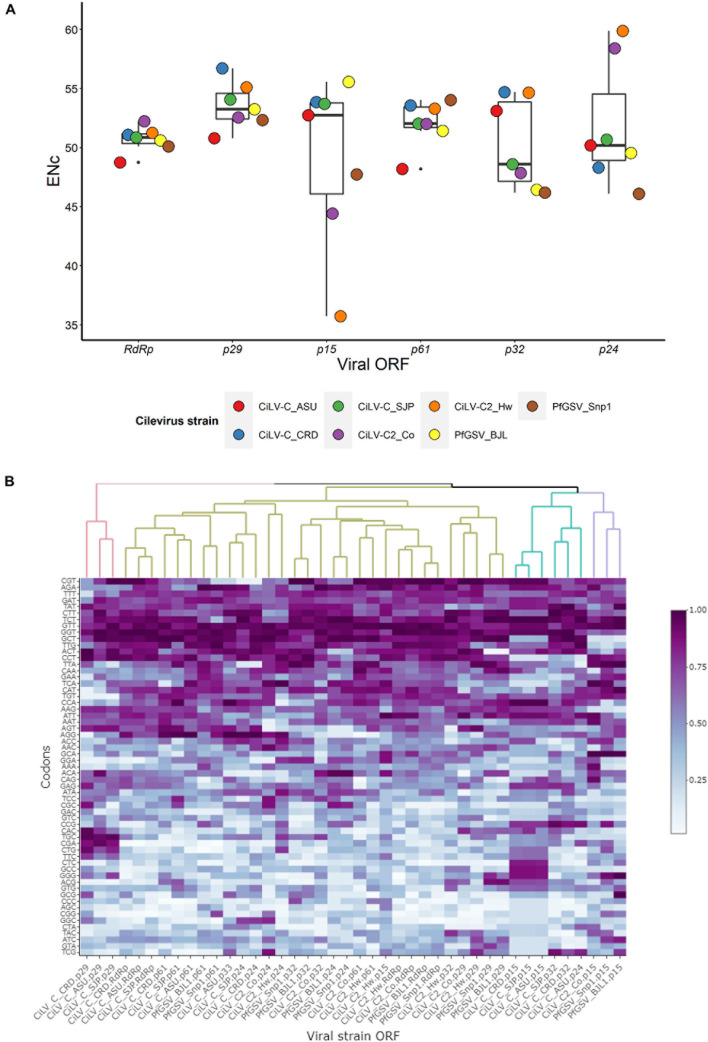
Codon usage bias of cilevirus ORFs. **(A)** Boxplot of Effective Number of codons (ENc). ENc values were calculated using COUSIN webserver (http://cousin.ird.fr/index.php) and plotted in Rstudio (Rstudio_team, 2021). **(B)** RSCU profiles across ORF of seven different isolates of three species of cileviruses. RSCU values were obtained using the CAIcal webserver (https://ppuigbo.me/programs/CAIcal/). The heat map was drawn in Rstudio using the “heatmapply” function (Galili et al., 2018). RSCU values were transformed using the “percentize” method and dist_method = “Euclidean” and hclust_method = “complete” were selected. Darker purple represents higher RSCU values.

Effective Number of codons values range from 20 to 61, with a value of 20 indicating extreme bias, whereas, inversely, 61 indicates non-biased codon usage (Wright, 1990). Meanwhile, an RSCU value higher or lower than 1 denotes that the codon has a positive or negative CUB, respectively, and those with an RSCU value equal to the unit are randomly chosen. In this study, ENc and RSCU values were calculated using the web servers COUSIN^1^ and CAIcal^2^, respectively. Analyses of both ENc and RSCU of the cilevirus ORFs revealed codon usage bias. Even though the means of the ENc values of ORFs both at virus and genus levels were generally higher than 50, some viral isolates and ORFs, e.g., PfGSV_Snp1 and ORF *p15*, had mean values lower than 50 which are considered an indicator of skewed codon usage. Within this group, the ORF *p15* of CiLV-C2_Hw displayed a distinctly low ENc value, ≈35 (Figure 4A and Supplementary Table 2), which is generally accepted as a mark of genes with significant codon usage bias (Comeron and Aguadé, 1998; Butt et al., 2016). The values of RSCU were computed for every codon of each ORF by the respective genomes (Supplementary Table 3). When the RSCU patterns were hierarchically clustered, most of the ORFs were grouped in a large branch of the generated dendrogram. ORFs *p15*, however, were generally distributed in two small branches shared also with the ORFs *p32* and *p24* of a few isolates (Figure 4B). Differently, the ORF *p15* of CiLV-C2_Hw was clustered together with the ORFs *RdRp* and *p61* of that isolate, suggesting that they could have undergone a comparable mutational selection.

To get further insight into the codon preferences of *p15*, we assessed and compared the CUB of each ORF in each cilevirus strain using COUSIN^1^ and the ratio RCDI/eRCDI^2^ (RCDI/expected RCDI) (Puigbò et al., 2010). It should be noted that while ENc and RSCU values exclusively rely on the coding sequence of the analyzed gene, COUSIN and the ratio RCDI/eRCDI assess the similarity of the codon usage patterns among the coding sequences of at least two genes. In this study, the COUSIN score (Bourret et al., 2019) estimates the codon usage preferences of a viral ORF compared with those of a reference, the viral host, normalized over a null hypothesis of equal usage of synonymous codons. Sequences with scores ≥ 1 display adaptation to the host cellular machinery and higher values might reflect an increasing level of expression. RCDI reflects the similarity of the codon usage between a given coding sequence and a reference genome (Mueller et al., 2006). A lower RCDI value shows the best rate of viral gene translation in the host as well as being indicative of the possible co-evolution of virus and host genomes. The eRCDI value is the RCDI corresponding to a random sequence generated with similar G + C content and amino acid composition to the analyzed sequence and provides a threshold that allows for statistical analysis of the calculated RCDI values. A lower ratio RCDI/eRCDI means a better viral ORF adaptation to its host.

The values of COUSIN and the ratio RCDI/eRCDI were calculated using the set of host plants whose codon usage data could be directly retrieved from, or calculated using available genomic information in, public databases. Codon usage frequencies of some natural and experimental hosts of cileviruses, i.e., citrus (*Citrus* × *sinensis*), passion fruit (*Passiflora* spp.), orchid (*Oncidium* spp.) (Kitajima et al., 2010), *Arabidopsis thaliana* (Arena et al., 2017), and *Phaseolus vulgaris* (Garita et al., 2013) were retrieved from Codon Usage Database^3^ or calculated using countcodon v4^4^ (Supplementary Table 4). Coding sequence information of *Passiflora edulis*, approximately 300 ORFs, was retrieved from the CoGe platform^5^. Orchids were included as cilevirus natural hosts since the infection by PfGSV of plants from two orchids species collected in Brazil was molecularly confirmed (unpublished results). For the sake of the comparisons, the analyses were performed considering both the sum of natural and experimental host plants and two subsets of plants, each comprising natural or experimental hosts. Altogether, the analysis included plants belonging to five families, including perennial woody (citrus) and perennial herbaceous (orchids) plants, short-lived evergreen perennial vine (passion fruit), and herbaceous annual (common beans and Arabidopsis) plants. Values of COUSIN and the ratio RCDI/eRCDI were visualized and compared using hierarchical clustering. The accuracy of the assessment could be potentially increased as new annotated genomes of cilevirus host plants are available.

**FIGURE 5:**
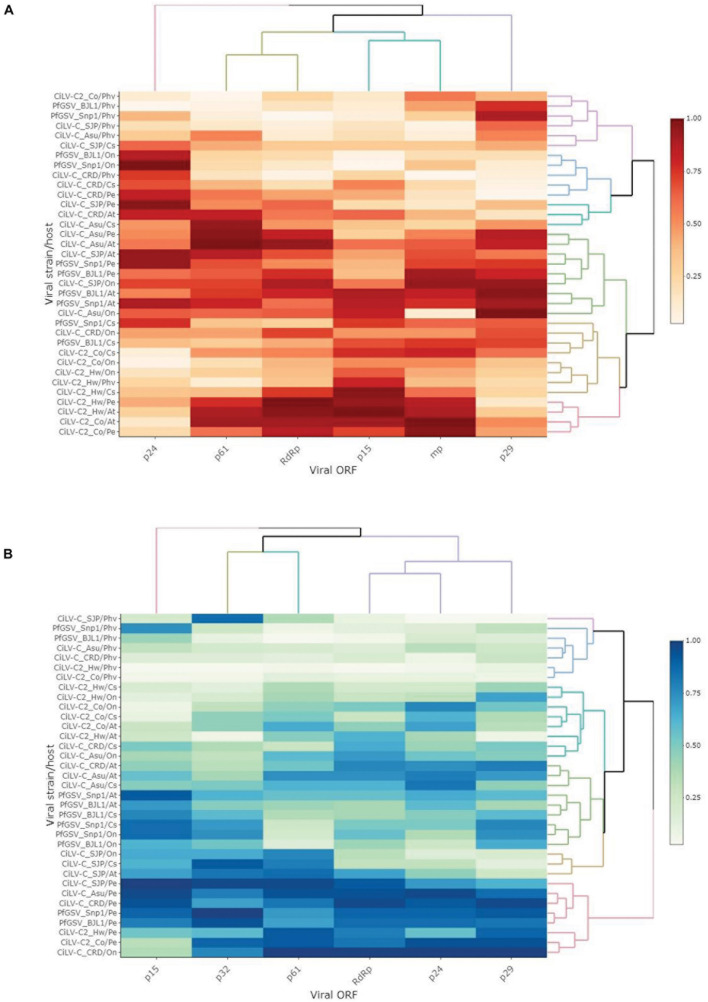
Codon adaption of cilevirus ORFs. Heatmaps representing the values of COUSIN **(A)** and the RCDI/eRCDI **(B)**, respectively, of every ORF across cilevirus isolates. For the sake of comparative analyses, COUSIN and the RCDI/eRCDI values have been obtained simulating the interaction of all viruses with the same hypothetical host range. Codon usage database of host plants and values of ENc, COUSIN, and RCDI are shown in Supplementary Tables 3, 4. The heat map was drawn in Rstudio (Rstudio_team, 2021) using the “heatmapply” function (Galili et al., 2018). Both COUSIN and RCDI/eRCDI values were transformed using the “percentize” method and dist_method = “Euclidean” and hclust_method = “complete” were selected. Darker red and light green to white represent the best values in each heat map, respectively. At, *Arabidopsis thaliana*; Cs, *Citrus* × *sinensis*; Pe, *Passiflora edulis*; On, *Oncidium* sp.; and Phv, *Phaseolus vulgaris*.

Regardless of the viral species, the analysis by ORFs using the complete set of host plants revealed a >1 bias in the mean COUSIN values of *p61* (1.28 ± 0.44), *RdRp* (1.19 ± 0.36), *p32* (1.15 ± 0.37), and *p24* (1.09 ± 0.38), whereas mean values below or near to 1 were observed for the ORFs *p15* (0.89 ± 0.53) and *p29* (0.93 ± 0.35) (Supplementary Table 5A). COUSIN values of *p15*, however, showed the highest variability with a coefficient of variation surpassing 50%. Some CiLV-C2 ORFs reached COUSIN values higher than 1.5, whereas the minimum values, e.g., −0.15, corresponded to PfGSV isolates. The same trend of COUSIN values was obtained when calculations were performed with the two subgroups of plants conveniently separated into natural and experimental hosts (Supplementary Tables 5B,C). In a global analysis across the genus, the COUSIN profile of *p15* resembled that showed by *p32*, which encodes the viral movement protein (Figure 5A).

The study of the ratio RCDI/eRCDI of the cilevirus ORFs indicated that *RdRp* and *p61* bear the lowest mean values, followed in ascending order by *p24*, *p29*, and *p32*, and finally *p15* (Supplementary Table 6A). In the particular comparisons of *p15*, values from CiLV-C2 were collectively the lowest ones reached. In the evaluations using *P. vulgaris*, the ratios RCDI/eRCDI of *p15* genes from CiLV-C2 isolates were below the threshold of 0.9, similar to those held by other viral ORFs, e.g., *p29*, *p24*, and *p32.* The analyses suggest that the CUB of all those ORFs might be the outcome of equivalent processes of strain/host adaptation.

Regardless of the subgroup of studied plants, i.e., the subsets of natural, experimental, or the array containing all host plants, the mean values of the ratio RCDI/eRCDI of *p15* were higher than those shown by the remaining viral ORFs (Supplementary Tables 6B,C). At the genus level, when analyzed through hierarchical clustering, the RCDI/eRCDI profile of *p15* is unique among those shown by any other ORF of cileviruses (Figure 5B).

## P15 Proteins Display a Unique Conserved Sequence Motif

Even though a low global sequence identity of less than 25% was observed for P15 proteins, the consensus sequence obtained from a multiple sequence alignment of the cysteine-rich domain reveals a stretch of highly conserved residues organized in the sequence motif {[FY]-L-x(3)-[FL]-H-x-[LIV]-S-C-x-C-x(2)-C-x-G-x-C} (Figure 3 and Supplementary Table 7). Besides four full-conserved residues of cysteines and one of histidine, the consensus sequence motif also contains three other invariable amino acids: leucine, serine, and glycine. A ScanProsite^6^ (de Castro et al., 2006) search using the conserved sequence motif in the UniProtKB/Swiss-Prot^7^ (release 07-Apr-2021: 564,638 entries) and UniProtKB/TrEMBL^8^ (release 07-Apr-2021: 214,406,399 entries) databases retrieved 30 hits spanned in 30 sequences, all of them exclusively corresponding to the P15 proteins from CiLV-C, CiLV-C2, and PfGSV. Additionally, no hits were retrieved from a similar search but against a randomized UniProtKB/Swiss-Prot database, i.e., reversed option. According to ScanProsite, the conserved sequence motif has an approximate number of expected random matches of 6.97 × 10^–08^ in 100,000 sequences (50,000,000 residues). Altogether, these results suggested a low probability of P15 matches by chance and, thus, highlighted the singular occurrence of the identified cysteine-rich domain in the P15 protein family.

## Three-Dimensional Structure Prediction Suggests a Helical Bundle-Like Scaffold for the P15 Proteins

The existence of a conserved sequence motif among the P15 proteins dropped a hint that all of them might exhibit a similar fold. Interestingly, this motif overlaps a predicted α-turn-α motif in the secondary structure of P15 in CiLV-C and CiLV-C2, whereas in PfGSV the second α-helix is missing (Figure 3). The predictions by evaluation of sequence-structure fitness (Ouzounis et al., 1993) with proteins of known 3D structures of a representative group of ten P15 proteins from the three species of cileviruses were carried out by the iterative threading assembly refinement (I-TASSER) server^9^ (Yang and Zhang, 2015). For the computational structural analyses, experimentally determined 3D structures were downloaded from the RCSB PDB database^10^ and the protein 3D structure alignments were done using the PDBeFold server^11^ (Krissinel and Henrick, 2004). The quality of protein structure templates and predicted full-length models were assessed using the *C*-score and TM-score implemented in I-TASSER^9^ (Zhang and Skolnick, 2004). *C*-score is typically in the range [−5,2], where a *C*-score of higher value signifies a model with high confidence, and vice-versa. TM-score values range from [0,1], where 1 indicates a perfect match between two structures. A TM-score below 0.17 corresponds to randomly chosen unrelated proteins whereas structures with a score higher than 0.5 assume generally the same fold.

**FIGURE 6:**
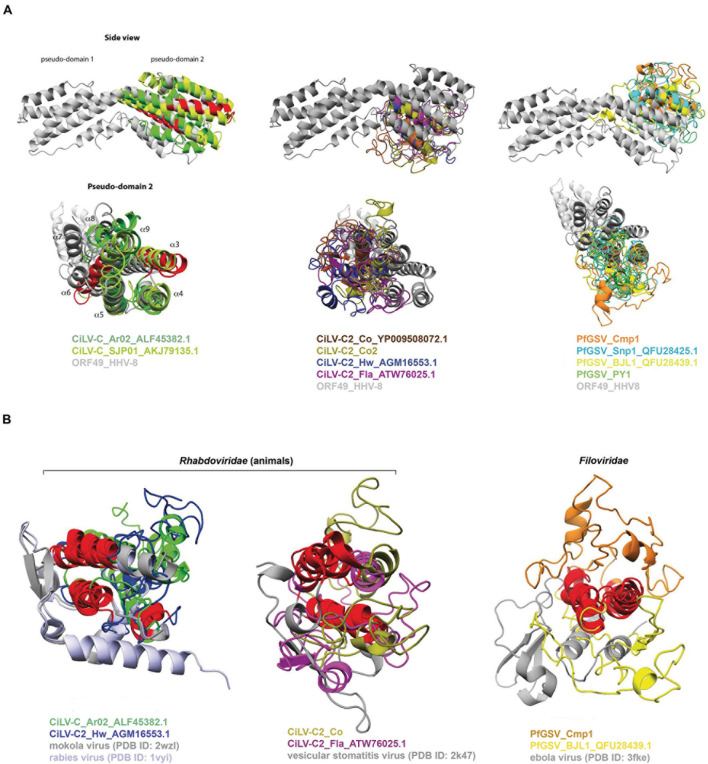
3D structural similarities between P15 and **(A)** the protein ORF49 from the human gammaherpesvirus 8 (ORF49_HHV-8, PDB ID: 5ipx) and **(B)** the rhabdoviruses rabies virus (PDB ID: 1vyi) and vesicular stomatitis Indiana virus (PDB ID: 2k47), and the filovirus Zaire ebolavirus (PDB ID: 3fke). Multiple structural alignments were done with the PDBeFold method. The conserved sequential motif {[FY]-L-x(3)-[FL]-H-x [LIV]-S-C-x-C-x(2)-C-x-G-x-C} is displayed in red. Structure figures were prepared using PyMOL (Delano, 2002).

The best generated P15 3D model corresponded to that for CiLV-C_Ar02 (*C*-score −2.82, TM-score 0.39 ± 0.13, Supplementary Table 8). The model from this protein suggested the presence of a four-helical bundle-like scaffold with structural similarity to the 12 α-helices bundled of protein ORF49 from the human gammaherpesvirus 8 (ORF49_HHV-8, PDB ID: 5ipx). The multiple structural alignments of 3D models of P15 proteins with the crystal structure of ORF49_ HHV-8 by using PDBeFOLD^11^ revealed the “plasticity” of the helix-bundle protein folds (Figure 6A).

PDBeFold provides average root mean square deviation (RMSD) and *Q*-score values to estimate the quality of matching structures. RMSD distance between corresponding residues is calculated after an optimal rotation of one structure to another. An RMSD of less than about 3–4 Å would generally be considered very close, but there is no absolute rule. The *Q*-score assesses the number of residues in the matched secondary structural elements and their positions in space. *Q*-scores reach the value of 1 for identical structures and decline to zero with the lack of similarity, thus, the higher *Q*, the better.

Among the P15 from CiLV-C isolates, an overall RMSD = 2.47 Å and *Q*-score = 0.31 over 94 aligned residues were obtained, whereas in those from CiLV-C2 the values were RMSD = 3.83 Å and *Q*-score = 0.01 over 22 aligned residues. In P15 from the PfGSV isolates, the values were an overall RMSD = 4.1 Å and *Q*-score = 0.02 over 30 aligned residues. Detailed analysis of the 3D model for the CiLV-C_Ar02 P15 indicated that cysteine residues at positions 85 (Cys85) and 92 (Cys92) could form a disulfide bridge and consequently stabilize the four-helical bundle. Remarkably, this two-cysteine-residues pattern is also present in the conserved sequence motif across the P15 proteins. However, since the spatial localization of the two cysteines is not close enough in some 3D models, the formation of a disulfide bridge is not completely evident. Such discrepancies could ensue from the analysis of low-resolution 3D models.

The resolved crystal structures of ORF49_ HHV-8 (PDB ID: 5ipx) and ORF49 from the murine herpesvirus 68 (ORF49_MHV68, PDB ID: 6a4v) (Hew et al., 2017; Chung et al., 2018) show a fold consisting of 12 α-helices bundled into two pseudo-domains (left top and bottom panels in Figure 6A). By mapping the identical and similar amino acids onto the helical scaffold, the potentially functional residues in P15 from CiLV-C were predicted. Conserved leucine and valine residues were localized in hydrophobic regions along the interacting surfaces of the α-helices and have the potential to play a structural role. Polar and charged residues were localized on the surface and could be associated with protein fold stability or protein interactions (Table 1).

**TABLE 1:** Summary of the predicted functional residues for the P15 family of proteins.

Amino acid^a^	ORF49^b^	3D^c^	Description
Cys 18	His 14	α1	Polar, exposed, protein interaction
Cys 20	Phe 16	α1	Polar, exposed, protein interaction
Asp 26	Ser 22	Loop	Charged, exposed, protein interaction
Leu 34	Leu 30	α2	Hydrophobic, buried, helix packing
Val 35	Asp 31	α2	Hydrophobic, buried, helix packing
Leu 50	Ala 46	α3	Hydrophobic, buried, helix packing
Leu 74	Pro 70	α3	Hydrophobic, buried, helix packing
Tyr 76	Leu 72	α3	Polar, exposed, protein interaction
His 79	Ala 75	α3	Charged, exposed, protein interaction
Ser 82	Ala 78	α3	Polar, exposed, protein interaction
Cys 83	Asn 79	α3	Polar, exposed, protein interaction
Asn 84	Leu 80	α3	Polar, exposed, protein interaction
Cys 85	Ala 81	α3	Polar, exposed, disulfide bridge, protein fold stability
Cys 88	Leu 84	α3	Polar, exposed, protein interaction
Gly 90	Gln 86	α3	Polar, exposed, protein fold stability
Cys 92	Tyr 88	α3	Polar, exposed, disulfide bridge, protein fold stability
Gly 98	Lys 94	α4	Polar, exposed, protein fold stability
Gly 114	Phe 113	α5	Polar, exposed, protein fold stability

*^*a*^Amino acid position in the CiLV-C_Ar02_ALF45382.1 sequence.*

*^*b*^Equivalent position in the ORF49 sequence according to the structure superposition between ORF49_HHV-8 (PDB ID: 5ipx) and CiLV-C_Ar02_ALF45382.1 (I-TASSER 3D-model).*

*^*c*^Nomenclature of alpha-helices is based on the 3D structure of ORF49_ HHV-8. The structure superposition between ORF49_HHV-8 and CiLV-C 3D-models by PDBeFold is provided in Supplementary File 1.*

α-helical folds have also been detected in the C-terminal domain of the phosphoprotein (P_*CTD*_) of mononegaviruses (Assenberg et al., 2010; Ivanov et al., 2010; Ng et al., 2020). It is of note that cileviruses and mononegaviruses of the genus *Dichorhavirus*, family *Rhabdoviridae* (Dietzgen et al., 2018), share several commonalities, including hosts and vectors, that have led to postulate the likely existence of evolutionary convergence among viruses of these two genera (Freitas-Astúa et al., 2018). The peculiar architecture observed in P_*CTD*_ and P15 prompted us to compare the 3D structure of these proteins.

The P_*CTD*_ of the cytorhabdovirus lettuce necrotic yellows virus (LNYV) comprises five α-helices that have an overall topology that although with different structural features, seems to be conserved with those in other rhabdoviruses, filoviruses, and paramyxoviruses (Martinez et al., 2013). P_*CTD*_ crystal structure from plant-infecting rhabdoviruses other than LNYV has not been described. Hence, the predicted 3D models of P15 were aligned with the P_*CDT*_ of mononegaviruses, regardless of their range hosts. P15 of CiLV-C_Ar02 and CiLV-C2_Hw are structurally related to P_*CTD*_ of mokola virus (PDB ID: 2wzl) (Delmas et al., 2010) and rabies virus (PDB ID: 1vyi) (Mavrakis et al., 2004) with overall RMSD = 3.1 Å and *Q*-score = 0.06 over 42 aligned residues, whereas those of CiLV-C2_Co2 and CiLV-C2_Fla are structurally related to vesicular stomatitis Indiana virus (PDB ID: 2k47) with overall RMSD = 4.0 Å and *Q*-score = 0.04 over 32 aligned residues (Figure 6B). In the case of PfGSV_Cmp1 and PfGSV_BJL1, their modeled P15 are structurally related to Zaire ebolavirus (PDB ID: 3fke) with an overall RMSD = 3.1 Å and *Q*-score = 0.04 over 36 aligned residues. Based on structural similarities, it could be hypothesized that P15 proteins can perform functions related to those accomplished by ORF49 and P_*CTD*_ proteins. ORF49 interacts and upregulates the transcriptional activity of a protein known as RTA (replication and transcription activator) in HHV-8, which drives the expression of all lytic genes (González et al., 2006). Present in a large number of viruses, P protein orthologs are involved in a wide range of functions, e.g., acting as chaperons, regulation of viral transcription, RNA binding, and suppressor of RNA silencing in plants (Masters and Banerjee, 1988; Peluso and Moyer, 1988; Martinez et al., 2013; Bejerman et al., 2016; Mann et al., 2016; Ng et al., 2020).

## Zn-Finger-Like Proteins in Arthropod-Infecting Viruses Closely Related to Kitaviruses Are Not Related With the Cilevirus P15 Proteins

Based on the predicted isoelectric point and the presence of a cysteine-rich pattern compatible with a potential Zn-finger domain, an acidic cilevirus P15-like protein was previously recognized in the genome of Wuhan insect virus 8 (WhIV-8) (Vinokurov and Koloniuk, 2019). The protein is encoded by the putative ORF *p11* (Figure 1) that was neither identified during the virus genome description (Shi et al., 2016) nor annotated at its corresponding GenBank locus KX883812.

Our search for supposed *p15* homologs based on the presence of a zinc-finger-like motif was expanded to nege/kita-like viruses and other related arthropod-infecting viruses. It also included previously ignored or poorly characterized putative ORFs. In members of the proposed subgroup Centivirus of the nege/kita-like viruses, a homolog of WhIV-8 *p11* was identified in the uncharacterized *Glycine dolichocarpa* virus (GDV, Transcriptomic shotgun assembly TSA GGIW01009300) (Kondo et al., 2020). Proteins encoded by the ORF *p11* in GDV and WhIV-8 have 41.4% amino acid sequence identity. The region comprising the cysteine-rich pattern in these two proteins shows a marginal identity (*E*-value < 1 according to MOTIF Search^12^) with the Pfam motif C4-type zinc-finger of DNA polymerase delta (PF14260). Similarly, the ORF p*19* in the putative centivirus Wuhan house centipede virus 1 (WHCV-1) and ORF *p9* of Hubei virga-like virus 9 (HVLV-9) encode proteins with signatures of a putative zinc-finger domain (Vinokurov and Koloniuk, 2019), which in the case of HVLV-9 also displays a subtle identity [*E*-value < 1 according to MOTIF Search^11^] with the Pfam motif SLBP (stem-loop binding protein) of the RNA binding superfamily (PF15247). The N-terminal of the ORF3-encoded protein of *Pyrrhocoris apterus* virus slightly resembles the canonical Pfam DSRM (double-stranded RNA binding motif, PF00035) architecture, whereas, although with low sequence similarity, the C-terminal end of this protein displays a conservative basic RNA-interacting motif. However, neither nucleotide sequences of these ORFs nor their predicted polypeptides show significant sequence identity to any element across cileviruses.

To get further insight into the putative relationships between P15 and P11 proteins of WhIV-8 and GDV, their 3D structures were predicted using the I-TASSER server^9^. Although P11 proteins show α helix scaffolds, the arrangement of helixes is different from that observed in P15. Multiple structural alignments of 3D models by PDBeFold^11^ indicated that only one α-helix in P11 proteins shows structural similarity to α-helices in the P15 3D models (overall RMSD = 2.68 Å and *Q*-score = 0.01 over 17 aligned residues). The lack of overall sequence or 3D structure similarity between both groups of proteins drastically reduces the probability of a common origin.

## The 5′-End of the RNA2 in Cileviruses, Besides the ORF *p15*, Harbors ORFs Encoding Small Hydrophobic Proteins

Downstream the ORF *p15*, the genomic stretch extended up to *p61* in the 5′-end of the RNA2 of cileviruses is known as the intergenic region (IR). However, this is not a proper description because rather than lacking ORFs, the IR lacks ORFs greater than 300 nucleotides. *In silico* tools have traditionally considered a minimum of 100 amino acids for an ORF to be annotated as a putative coding protein sequence, which has led to the inaccurate annotation of certain genomes. However, despite the detection of those small ORFs, the IRs of cileviruses show a very low density of predicted coding sequences, only 20–30%, with large non-coding stretches (Figure 1).

In congruence with ORF *p15*, the interspecies comparisons of the IRs of cileviruses show low levels of nucleotide sequence identity (≤45%) (Ramos-González et al., 2020). With variable length, the IR is larger in CiLV-C isolates (1,095 ± 56 nucleotides) and shorter in PfGSV isolates (850 ± 21 nucleotides). The region harbors small ORFs encoding hydrophobic proteins with predicted TM domains, but which are unrelated to the SP24 motif (Melzer et al., 2013; Roy et al., 2013; Ramos-González et al., 2016, 2020). ORF *p13* in PfGSV_Snp1, the largest detected so far (333 nucleotides), presents four regularly distributed cysteine residues and low sequence identity with motifs identified in the cytokine-induced anti-apoptosis inhibitor 1 (CIAPIN1, PF05093), and PGC7/Stella/Dppa3-like domain (PGC7_Stella, PF15549) (Ramos-González et al., 2020).

Further inspection in the 5′-end of the RNA2 in cileviruses, allowed us to detect the ORF *p8* overlapping the ORF *p15* of CiLV-C. ORF *p8* is present in every sequenced virus of the three lineages of CiLV-C (Chabi-Jesus et al., 2021), but it is absent in CiLV-C2 and PfGSV. Previously overlooked, the ORF *p8* spans 54% of *p15* and has 213 nucleotides in the frame +2 (Figure 1). ORF *p8* is significantly longer than any other ORF expected in all reading frames within the ORF *p15*, which is a fair indicator of its real existence according to an *in silico* method for detecting overlapping genes (Schlub et al., 2018) performed in this study (Supplementary Table 9). The presence of the overlapping ORF *p8* in CiLV-C may limit the variability of *p15* and suggests a different evolutionary trajectory in the formation of this genomic region among cileviruses.

The predicted P8 protein seems to be hydrophobic with a TM domain according to MEMSAT-SVM^13^ and TMHMM Server v. 2.0^14^. This TM domain resembles the predicted properties of the small P7 proteins encoded downstream the ORF *p15*, in the IR of CiLV-C2 (Roy et al., 2013), CiLV-C_SJP, and PfGSV (Figure 1). Small hydrophobic proteins by themselves are outside the scope of this work but what is particularly pertinent is their constant presence in the 5′-end of the RNA2 of cileviruses, and the association with the small Zn-finger-like or nucleic acid binding-like proteins in some nege/kita-like viruses. The overlapping gene array observed in the pair P15/P8 in CiLV-C was also observed in the pairs P11/P6 and P19/P8 of the centiviruses WhIV-8 and WHCV-1, respectively (Figure 1). Overlapping genes *p6* and *p8* of centiviruses were detected following the same procedure described for the identification of *p8* in CiLV-C (Supplementary Table 9). Small hydrophobic proteins have been described in several taxonomic groups of plant RNA viruses where they can be involved in coupling viral replication and cell-to-cell movement showing properties similar to those of reticulons (Solovyev and Morozov, 2017; Morozov and Solovyev, 2020; Lazareva et al., 2021).

## Discussion

This study was driven by the search for commonalities between the ORFs *p15*, their encoded proteins, and the questioning of whether these polypeptides can be considered orthologs. Finally, these analyses led us to a critical appraisal of the 5′-end of the RNA2 of cileviruses under an evolutionary perspective.

Absent in kitavirids beyond the genus *Cilevirus* and with no apparent homology with any sequence available in public databases, ORF *p15* was considered an orphan gene (Arena et al., 2016). P15 proteins from different species show a remarkably reduced amino-acid sequence identity between them, with values as low as those shown by the global alignment of unrelated proteins (≈16%) (Konaté et al., 2019; Ramos-González et al., 2020). As well as P13 from PfGSV, it has been speculated that P15 proteins are viral auxiliary factors that act as regulators of host physiology during the plant-virus interplay (Ramos-González et al., 2020). Particularly, the putative role of P15 from CiLV-C of the strain CRD as a viral suppressor of the plant gene silencing antiviral defense was analyzed (Leastro et al., 2020), but further studies are needed to reach sound conclusions.

Together with the hallmark ORF *RdRp* (Koonin et al., 2006), the genomes of the typical cileviruses comprise the ORFs *p24, p32*, and the taxonomically restricted *p61.* Their RNA2 molecules also accommodate at their 5′-ends the *p15* and the IR, which in addition to *p11* and *p13* in PfGSV strains, harbor some putative small ORFs encoding predicted TM small polypeptides. Interestingly, hibiscus yellow blotch virus (HYBV) (Olmedo-Velarde et al., 2021), a specimen considered a cilevirus with an unusual genomic organization and phylogenetically intermediate among typical cileviruses and hibiscus green spot virus 2 (HGSV-2), genus *Higrevirus* (Melzer et al., 2012), poses an additional puzzle on the composition of the RNA2 segments of typical cileviruses and the role of the ORFs in their 5′-ends, e.g., *p15*. While the RNA1 in all these viruses encodes two proteins, the RdRp and P29 or P10 in typical cileviruses and HYBV, respectively, the RNA2 of HYBV resembles a truncated version of the RNA2 of typical cileviruses, lacking the 5′- end stretch upstream the homologs of the threesome *p61*-*p32*-*p24* (Figure 1). RNA2 of HYBV also includes an additional ORF, homolog to the cilevirus RNA1 *p29*, at its 3′-end. The very conserved synteny and composition among the genomes of HYBV and typical cileviruses suggest, under a reductionist approach, the rather dispensable role of the 5′-end of the RNA2 of cileviruses. Perhaps, this region only provides proteins and/or unknown regulatory non-coding RNAs that albeit non-essential could expand the viral fitness (Frías-Lasserre, 2012; Miller et al., 2016). Alternatively, we could speculate, for instance, that the putative functions encoded by this genomic region in typical cileviruses are taken over by other ORFs in HYBV, likely involving a compensatory evolutionary process (Rokyta et al., 2002; Harcombe et al., 2009). However, while the lack of function of the 5′ end of RNA2 in typical cileviruses does not appear to be a rational choice, in the absence of compelling evidence suggesting explicit roles, the analysis of *p15* across the genus, intrinsically, may shed some light on the 5′-end of cilevirus RNA2.

In this study, we described that *p15* in each of the three cilevirus species is under purifying selection, suggesting an ongoing action to conserve its nucleotide sequence, most likely precluding the change of amino acid residues and preserving function at directly selected sites (Cvijović et al., 2018). Furthermore, we detected that in some viral strains the synonymous codon usage of this ORF is biased showing the same codon patterns adopted by other viral genes and displaying COUSIN and RCDI values that suggest a certain level of adaptation to its plant hosts. We also revealed that although with extremely divergent amino acid sequences, P15 proteins show a conserved sequence motif, and their 3D models have preserved a backbone topology that resembles a helical bundle-like scaffold. Altogether, these two aspects suggest elements of common ancestry and conserved functionality of the P15 proteins (Arendsee et al., 2019; Ng et al., 2020; Ravantti et al., 2020).

The superimposition of the 3D models of P15 and the resolved crystal structures of ORF49_ HHV-8 and ORF49_MHV68 (Hew et al., 2017; Chung et al., 2018) and the P_*CTD*_ of negative-stranded RNA viruses of the order *Mononegavirales* (Martinez et al., 2013) indicated the presence of an α-helical bundle-like scaffold in the cilevirus protein. Bundles of four α-helices are frequently found in proteins with a wide variety of functions (Rhys et al., 2019). ORF49 shows a fold consisting of 12 helices bundled into two pseudo-domains, with charged patches on its surface likely acting in protein-protein interaction sites. The protein is stabilized by double-stranded oligonucleotides suggesting its interaction and binding with DNA molecules (Hew et al., 2017). The P_*CTD*_ of negative-stranded RNA viruses of the order *Mononegavirales* (Martinez et al., 2013) has been shown to regulate viral transcription, RNA binding, and immunomodulatory functions (Ng et al., 2020). The P protein of cytorhabdoviruses has weak local RNA silencing suppressor activity, strongly suppresses systemic RNA silencing, and the P_*CTD*_ is essential for both local RNA silencing suppression and the interaction with AGO1, AGO2, AGO4, RDR6, and SGS3, proteins of the main core of the gene silencing mechanism (Bejerman et al., 2016; Mann et al., 2016). Therefore, the presence of an α-helical bundle-like scaffold in P15 could provide a structural base by mean it could interact with other proteins and likely mediate the plant-virus interplay. P15 from CiLV-C of the strain CRD forms homo- and heterodimers with other viral proteins (Leastro et al., 2018), and considering the diversity of its interactions, it appears that the protein could act modulating a wide spectrum of viral and cellular pathways, as observed in other viral proteins (Deblasio et al., 2018; Dao et al., 2020; Li et al., 2020).

### The Origin and Evolution of the 5′-End of Cileviruses: A Theory

Phylogenetic inferences suggest a common ancestor between kitaviruses and a variety of arthropod-infecting viruses including negeviruses (Vasilakis et al., 2013; Kondo et al., 2019; Ramos-González et al., 2020; Quito-Avila et al., 2021). Having regards to the presence of the orthologs *RdRp* and *p24* in these viruses (Kuchibhatla et al., 2014), a synteny-based analysis indicates a parsimonious evolutionary scenario in which cileviruses, and kitaviruses in general, derived from a monopartite ss(+) RNA through genome segmentation. In brief, the process could have started amid assortments of defective molecules resulting from error-prone replication and recombination events (Bangham and Kirkwood, 1993; García-Arriaza et al., 2004; Vignuzzi and López, 2019) and sub-genomic RNA transcripts derived from the ancestor. Those molecules with self-replication activity could have recruited segments supplying viral complementary functions. *Cis*- and *trans*-acting sequence motifs likely evolved providing these novel genomic segments with the capacity to coordinate essential processes for the persistence of split viral genomes, e.g., multiplication and packaging (Newburn and White, 2015). Further modifications of kitavirus genomes likely included the acquisition of auxiliary genes, as seems to be a general pathway in the formation of plant virome (Dolja et al., 2020). The clearest examples of horizontal gene transfer in the genome of current kitaviruses are epitomized by the ORF *p32* in cileviruses, and ORFs *BMB1* and *2*, which encode the binary movement block proteins in HGSV-2 (Quito-Avila et al., 2013; Solovyev and Morozov, 2017; Hao et al., 2018).

Multiplication of unnecessary viral sequences appears to have fitness costs and the size of virus genomes shows a natural trend to shrinkage (Belshaw et al., 2007; Zwart et al., 2014). The occurrence of IRs as long as observed in the RNA2 of cileviruses is atypical. Whether large IRs existed in the ancestors of kita/nege-like viruses, they were most likely purged by selection. The genomic organization of known negeviruses and kita/nege-like viruses are rather compact, with short stretches of intergenic sequences. Therefore, a relevant interrogation in this context is whether the IRs of cileviruses deviate from the selection rules. In a sense, most likely not, and some features might suggest it. The IR of PfGSV isolates are shorter and contain more predicted coding regions than those in CiLV-C isolates, whereas the presence of the overlapping ORF *p8* in CiLV-C may also indicate genomic compression.

As defined in this study, the 5′-end of the RNA2 genomic segments in typical cileviruses comprises the ORF *p15* and the IR. There is no obvious evidence allowing for a direct connection between the 5′-end of the cilevirus RNA2 molecules and any genetic element in the genome of their closely related negeviruses and nege/kita-like viruses. Yet, the relationship between the 5′-end of the RNA2 molecules from cileviruses would be difficult to establish, except for the presence of a conserved amino acid motif across the P15 family and the 3D structure similarities among these proteins. These two features, moreover, may be pertinent clues suggesting the common ancestry of the ORF *p15* present in the cileviruses CiLV-C, CiLV-C2, and PfGSV, giving support to a theory about their common origin.

The origin of the 5′-end of the RNA2 in typical cileviruses could have involved the following plausible scenarios: (*i*) a reminiscent ancient ORF from the monopartite ancestor or (*ii*) its acquisition from a heterologous source by recombination, i.e., horizontal gene transfer. Although we lack evidence to exclude any of these possibilities, our approach using prediction and computational analysis of 3D protein structures has provided information on a possible external origin of P15 proteins. The scaffold of α-helixes detected in P15 shows structural resemblance with those detected in well-characterized proteins from herpesvirids and mononegavirids. Importantly, the odds of the 3D structure of P15 is the result of convergence are very low considering the small number of protein folds available (Malik et al., 2017).

The external origin of the ORF *p15* could be considered part of the horizontal gene transfer events that likely happened in the course of kitavirus evolution, but what is peculiar in this case is the participation of a mononegavirid, probably an ancestor of the current plant rhabdoviruses, as its putative donor. Cileviruses and plant-infecting rhabdoviruses have a partially shared plant host range, and, in the case of dichorhaviruses, they also share mite vectors (Dietzgen et al., 2018, 2020; Freitas-Astúa et al., 2018). Likely, *Brevipalpus* mites, where dichorhavirus also multiplicate, have underpinned a primordial and essential role in the segmentation of both cileviruses and dichorhaviruses (Kondo et al., 2017).

However, whatever the scenario for the origin of *p15* in cileviruses, the very conservation of a sequence motif and fold in the encoded proteins indicate that the “ancestor” ORF was present before the radiation of, at least, CiLV-C, CiLV-C2, and PfGSV. Since then, it has been likely undergone a transformation process under selective pressure, acquiring or losing protein secondary structure elements and involving host-specific patterns to provide evolutionarily competitiveness to these viruses under new ecological conditions, e.g., the change of natural host range from arthropods to plants. Indeed, if this would be the case, *p15* likely became a fitness factor whose fixation might have indirectly contributed to the conservation of the IR. This large chunk of genomic sequence with apparently an unclear role is preserved between functional ORFs, that away of inactivity, it may be a zone of gain and loss of start and stop codons gradually incorporating novel ORFs.

Two mechanisms explaining the origin of orphan genes are currently accepted: (*i*) divergence from the coding sequence of a preexisting gene (Tautz and Domazet-Lošo, 2011; Vakirlis et al., 2020), and (*ii*) *de novo* appearance from intergenic or non-genic regions (Carvunis et al., 2012; Van Oss and Carvunis, 2019). The irregular organization and lack of identity among the small ORFs other than *p15* found in the 5′-end of the RNA 2 of cileviruses support the idea that this genomic region might act as “the cradle of new genes” (Forterre and Gaïa, 2016). Several of the predicted ORFs found in the IR are small, of ≈200 nucleotides, and they encode putative proteins where TM domains are detected by *in silico* analyses. Viral miniproteins with TM domains are encoded by a wide range of DNA and RNA viruses and they can bind and modulate cellular transmembrane proteins, providing flexible mechanisms to regulate several cellular activities (Dimaio, 2014; Finkel et al., 2018). Moreover, small ORFs can act as seeds for larger coding sequences (CDS) through a mechanism known as “CDS elongation” when their stop codons undergo mutations (Couso and Patraquim, 2017). Thus, small ORFs could provide an extra coding capacity that in the course of evolution could eventually become an additional source of fitness (Guerra-Almeida and Nunes-da-Fonseca, 2020).

## Concluding Remarks

In this study, we have addressed one of the most intriguing features of the molecular biology of cileviruses: the 5′ end of the RNA2 molecule and its two most salient elements, the ORF *p15*, and the IR. We presented pieces of evidence on the putative biochemical activity of the protein P15 over the genus and elucubrate on its putative contribution to viral fitness. The results are supported by the analyses of 58 nucleotide sequences of isolates of the three known species whose P15 amino acid sequences cover a spectrum of identity ranging from 14 to 100%. The main predictions have been obtained by integrating the results of different computational methods because of the premise that each of these elements taken in isolation does not allow to draw solid conclusions about the activity and function of a given protein (Omer et al., 2017; Ponting, 2017; Ravantti et al., 2020). Taken them together, we posit a theory that partially describes the evolution of the cilevirus genomes that involves elements denoting the likely close interplay during the coevolutionary history of *Brevipalpus*-transmitted viruses. In light of the plausible scenario described by the theory, two aspects of the biology of cileviruses gain new distinctions: (*i*) since *p15* across cileviruses can be considered alleles descendant from a common ancestor likely co-opted from another virus, this ORF should not be longer considered an orphan gene, and (*ii*) the markedly chimeric origin of the RNA2 genome segment, which adds further support to what appears to be a common feature of members of the family *Kitaviridae* (Morozov et al., 2020).

## Data Availability Statement

The original contributions presented in the study are included in the article/Supplementary Material, further inquiries can be directed to the corresponding authors.

## Author Contributions

PR-G and TP: conceptualization. PR-G, TP, CC-J, and GA: formal analysis, investigation, and methodology. JF-A: funding acquisition. TP and JF-A: resources. PR-G and TP: writing—original draft. PR-G, TP, CC-J, GA, and JF-A: writing—review and editing. All authors approved the submitted version.

## Conflict of Interest

The authors declare that the research was conducted in the absence of any commercial or financial relationships that could be construed as a potential conflict of interest.

## Publisher’s Note

All claims expressed in this article are solely those of the authors and do not necessarily represent those of their affiliated organizations, or those of the publisher, the editors and the reviewers. Any product that may be evaluated in this article, or claim that may be made by its manufacturer, is not guaranteed or endorsed by the publisher.

## Footnotes

1
http://cousin.ird.fr/index.php

2
https://ppuigbo.me/programs/CAIcal/

3
http://www.kazusa.or.jp/codon/

4
http://www.kazusa.or.jp/codon/countcodon.html

5
https://genomevolution.org/coge/

6
https://prosite.expasy.org/scanprosite/

7
https://www.uniprot.org/statistics/Swiss-Prot

8
https://www.uniprot.org/statistics/TrEMBL

9
https://zhanglab.ccmb.med.umich.edu/I-TASSER/

10
https://www.rcsb.org

11
https://www.ebi.ac.uk/msd-srv/ssm/

12
https://www.genome.jp/tools/motif/

13
http://bioinf.cs.ucl.ac.uk/psipred/

14
http://www.cbs.dtu.dk/services/TMHMM/
