# Supplementary material for: Poorly Conserved P15 Proteins of Cileviruses Retain Elements of Common Ancestry and Putative Functionality: A Theoretical Assessment on the Evolution of Cilevirus Genomes
Source: Front Plant Sci. 2021 Nov 5;12:771983. doi: 10.3389/fpls.2021.771983 (PMC8602818; doi:10.3389/fpls.2021.771983)
Supplement: Supplementary file 1 [file Data_Sheet_1.PDF]

```
PDBeFold v2.59. (src3) 14 Apr 2014 result file.

                  RESULTS OF MULTIPLE ALIGNMENT

 SUMMARY

                                                      Consensus scores
 ##    Structure                        Nres  Nsse   RMSD  Q-score
  1  5ipx.pdb:A  .....................   282   13   1.4246  0.1013
  2  model1_p15_CiLV-C_Ar02_ALF453>...   130    7   1.6586  0.2062
  3  model1_p15_CiLV_C_SJP.pdb:A  ....   130    4   1.6014  0.2095

Number of aligned residues 35
Number of aligned SSEs     2
Overall RMSD               2.7101
Overall Q-score            0.0184


 SECONDARY STRUCTURE ALIGNMENT

5ipx.pdb:A  ...........  -hshhHHhhhhhhh
model1_p15_CiLV-C_A>...  hhhhhHH-------
model1_p15_CiLV_C_S>...  -----HHhh-----


 SECONDARY STRUCTURES DETAILS

5ipx.pdb:A  ......                             |h|a:phe  12 /arg  17 | ==>
model1_p15_CiL>... |h|a:ile   7 /cys  18 | ==> |h|a:glu  24 /thr  29 | ==>
model1_p15_CiL>...

5ipx.pdb:A  ...... |s|a:tyr  18 /tyr  20 | ==> |h|a:ser  22 /asp  31 | ==>
model1_p15_CiL>... |h|a:asp  32 /his  56 | ==> |h|a:leu  57 /pro  61 | ==>
model1_p15_CiL>...

5ipx.pdb:A  ...... |h|a:cys  42 /leu  90 | ==> |H|A:ASN  92 /GLY 109 | ==>
model1_p15_CiL>... |h|a:asp  62 /asp  86 | ==> |H|A:THR  96 /PHE 113 | ==>
model1_p15_CiL>...                             |H|A:ASN   3 /PHE  21 | ==>

5ipx.pdb:A  ...... |H|A:GLY 112 /TYR 129 | ==> |h|a:cys 144 /ser 150 | ==>
model1_p15_CiL>... |H|A:GLY 114 /THR 130 | ==>
model1_p15_CiL>... |H|A:ASP  32 /VAL  58 | ==> |h|a:pro  61 /lys  87 | ==>

5ipx.pdb:A  ...... |h|a:ser 156 /thr 168 | ==> |h|a:ser 177 /leu 190 | ==>
model1_p15_CiL>...
model1_p15_CiL>... |h|a:thr  95 /thr 130 | ==>

5ipx.pdb:A  ...... |h|a:asp 201 /glu 227 | ==> |h|a:gln 228 /arg 256 | ==>
model1_p15_CiL>...
model1_p15_CiL>...
```

```
5ipx.pdb:A  ...... |h|a:ser 261 /ala 274 | ==> |h|a:ser 280 /phe 299 |
model1_p15_CiL>...
model1_p15_CiL>...


 CROSS-STRUCTURE STATISTICS


<< RMSD >>
  Structure                 1       2       3
------------------------+-------+-------+-------
  1. 5ipx.pdb:A  ......           2.645   2.537
  2. model1_p15_CiL>...   2.645           2.933
  3. model1_p15_CiL>...   2.537   2.933


<< Q-score >>
  Structure                 1       2       3
------------------------+-------+-------+-------
  1. 5ipx.pdb:A  ......           0.019   0.019
  2. model1_p15_CiL>...   0.019           0.037
  3. model1_p15_CiL>...   0.019   0.037


<< Sequence Identity >>
  Structure                 1       2       3
------------------------+-------+-------+-------
  1. 5ipx.pdb:A  ......           0.114   0.057
  2. model1_p15_CiL>...   0.114           0.029
  3. model1_p15_CiL>...   0.057   0.029


 ROTATION-TRANSLATION MATRICES OF BEST SUPERPOSITION

                   |   0.99991   0.01365   0.00088 |   | x |   |   0.13924 |
5ipx.pdb:A  ...... |  -0.01363   0.99973  -0.01883 | * | y | + |   0.66213 |
                   |  -0.00114   0.01882   0.99982 |   | z |   |   0.14658 |

                   |  -0.74950  -0.55689   0.35795 |   | x |   |  27.88531 |
model1_p15_CiL>... |  -0.63039   0.76547  -0.12907 | * | y | + | -15.79530 |
                   |  -0.20212  -0.32238  -0.92478 |   | z |   | 115.05282 |

                   |   0.93275  -0.36039  -0.00997 |   | x |   | -53.43226 |
model1_p15_CiL>... |   0.01064   0.05518  -0.99842 | * | y | + |  41.04643 |
                   |   0.36037   0.93117   0.05530 |   | z |   | -42.20316 |


 3D ALIGNMENT OF BACKBONE ATOMS
```

##		5ipx.pdb		model1_p15_C	model1_p15_C
1				a:met 1	
2				a:leu 2	
3				a:asn 3	
4				a:trp 4	
5				a:ser 5	
6				a:thr 6	
7			h	a:ile 7	
8			h	a:glu 8	
9			h	a:trp 9	
10			h	a:asp 10	
11			h	a:ser 11	
12			h	a:phe 12	
13			h	a:trp 13	
14		a:his 10	h	a:gln 14	
15		a:leu 11	h	a:gln 15	
16	h	a:phe 12	h	a:his 16	
17	h	a:asn 13	h	a:asp 17	
18	h	a:his 14	h	a:cys 18	
19	h	a:leu 15		a:gly 19	
20	h	a:phe 16		a:cys 20	
21	h	a:arg 17		a:phe 21	
22	s	a:tyr 18		a:thr 22	
23	s	a:his 19		a:phe 23	
24	s	a:tyr 20	h	a:glu 24	
25		a:pro 21	h	a:cys 25	
26	h	a:ser 22	h	a:asp 26	
27	h	a:trp 23	h	a:phe 27	
28	h	a:asp 24	h	a:ile 28	
29	h	a:gln 25	h	a:thr 29	
30	h	a:ile 26		a:ser 30	
31	h	a:leu 27		a:ile 31	
32	h	a:gln 28	h	a:asp 32	
33	h	a:glu 29	h	a:pro 33	
34	h	a:leu 30	h	a:leu 34	
35	h	a:asp 31	h	a:val 35	
36		a:thr 32	h	a:his 36	
37		a:leu 33	h	a:asp 37	
38		a:ser 34	h	a:tyr 38	
39		a:val 35	h	a:ala 39	
40		a:ala 36	h	a:ile 40	
41		a:thr 37	h	a:tyr 41	
42		a:leu 38	h	a:his 42	
43		a:asn 39	h	a:ser 43	
44		a:pro 40	h	a:leu 44	
45		a:asp 41	h	a:ser 45	
46	h	a:cys 42	h	a:gln 46	
47	h	a:his 43	h	a:lys 47	

```
48 |h|a:val  44 | |h|a:thr  48 | |            |
49 |h|a:pro  45 | |h|a:val  49 | |            |
50 |h|a:ala  46 | |h|a:leu  50 | |            |
51 |h|a:leu  47 | |h|a:glu  51 | |            |
52 |h|a:asn  48 | |h|a:met  52 | |            |
53 |h|a:val  49 | |h|a:leu  53 | |            |
54 |h|a:glu  50 | |h|a:gln  54 | |            |
55 |h|a:lys  51 | |h|a:thr  55 | |            |
56 |h|a:thr  52 | |h|a:his  56 | |            |
57 |h|a:leu  53 | |h|a:leu  57 | |            |
58 |h|a:tyr  54 | |h|a:val  58 | |            |
59 |h|a:leu  55 | |h|a:ala  59 | |            |
60 |h|a:ala  56 | |h|a:gly  60 | |            |
61 |h|a:lys  57 | |h|a:pro  61 | |            |
62 |h|a:thr  58 | |h|a:asp  62 | |            |
63 |h|a:ile  59 | |h|a:ala  63 | |            |
64 |h|a:gln  60 | |h|a:ser  64 | |            |
65 |h|a:ile  61 | |h|a:glu  65 | |            |
66 |h|a:leu  62 | |h|a:thr  66 | |            |
67 |h|a:val  63 | |h|a:ile  67 | |            |
68 |h|a:gln  64 | |h|a:arg  68 | |            |
69 |h|a:his  65 | |h|a:arg  69 | |            |
70 |h|a:arg  66 | |h|a:gln  70 | |            |
71 |h|a:gln  67 | |h|a:val  71 | |            |
72 |h|a:ser  68 | |h|a:ala  72 | |            |
73 |h|a:glu  69 | |h|a:phe  73 | |            |
74 |h|a:pro  70 | |h|a:leu  74 | |            |
75 |h|a:tyr  71 | |h|a:ile  75 | |            |
76 |h|a:leu  72 | |h|a:tyr  76 | |            |
77 |h|a:val  73 | |h|a:asp  77 | |            |
78 |h|a:pro  74 | |h|a:phe  78 | |            |
79 |h|a:ala  75 | |h|a:his  79 | |            |
80 |h|a:ala  76 | |h|a:arg  80 | |            |
81 |h|a:arg  77 | |h|a:leu  81 | |            |
82 |h|a:ala  78 | |h|a:ser  82 | |            |
83 |h|a:asn  79 | |h|a:cys  83 | |            |
84 |h|a:leu  80 | |h|a:asn  84 | |            |
85 |h|a:ala  81 | |h|a:cys  85 | |            |
86 |h|a:tyr  82 | |h|a:asp  86 | |            |
87 |h|a:ser  83 | | |a:lys  87 | |            |
88 |h|a:leu  84 | | |a:cys  88 | |            |
89 |h|a:gln  85 | | |a:cys  89 | |            |
90 |h|a:gln  86 | | |a:gly  90 | |            |
91 |h|a:leu  87 | | |a:his  91 | | |a:met   1 |
92 |h|a:tyr  88 | | |a:cys  92 | | |a:leu   2 |
93 |h|a:lys  89 | | |a:asn  93 | |h|a:asn   3 |
94 |h|a:leu  90 | | |a:ala  94 | |h|a:trp   4 |
95 | |a:gly  91 | | |a:thr  95 | |h|a:ser   5 |
96 |H|A:ASN  92 |*|H|A:THR  96 |*|H|A:THR   6 |
97 |H|A:ASP  93 |*|H|A:THR  97 |*|H|A:ILE   7 |
```

98	H	A:LYS 94	*	H	A:GLY 98	*	H	A:GLU 8
99	H	A:ILE 95	*	H	A:ARG 99	*	H	A:TRP 9
100	H	A:ARG 96	*	H	A:PHE 100	*	H	A:ASP 10
101	H	A:GLY 97	*	H	A:LYS 101	*	H	A:SER 11
102	H	A:VAL 98	*	H	A:VAL 102	*	H	A:PHE 12
103	H	A:ILE 99	*	H	A:VAL 103	*	H	A:TRP 13
104	H	A:ASN 100	*	H	A:ASP 104	*	H	A:GLN 14
105	H	A:GLY 101	*	H	A:ARG 105	*	H	A:GLN 15
106	H	A:MET 102	*	H	A:VAL 106	*	H	A:HIS 16
107	H	A:LEU 103	*	H	A:LEU 107	*	H	A:ASP 17
108	H	A:PRO 104	*	H	A:ASN 108	*	H	A:CYS 18
109	H	A:LEU 105	*	H	A:ASP 109	*	H	A:GLY 19
110	H	A:VAL 106	*	H	A:HIS 110	*	H	A:CYS 20
111	H	A:ASP 107	*	H	A:ILE 111	*	H	A:PHE 21
112								a:thr 22
113								a:phe 23
114								a:glu 24
115	H	A:ALA 108	*	H	A:GLU 112	*		A:CYS 25
116	H	A:GLY 109	*	H	A:PHE 113	*		A:ASP 26
117		a:cys 110						a:phe 27
118		a:ile 111						a:ile 28
119								a:thr 29
120								a:ser 30
121								a:ile 31
122							h	a:asp 32
123							h	a:pro 33
124							h	a:leu 34
125							h	a:val 35
126							h	a:his 36
127	h	a:gly 112					h	a:asp 37
128	H	A:PHE 113	*	H	A:GLY 114	*	H	A:TYR 38
129	H	A:GLU 114	*	H	A:ILE 115	*	H	A:ALA 39
130	H	A:ARG 115	*	H	A:MET 116	*	H	A:ILE 40
131	H	A:GLU 116	*	H	A:ARG 117	*	H	A:TYR 41
132	H	A:LEU 117	*	H	A:ARG 118	*	H	A:HIS 42
133	H	A:ILE 118	*	H	A:GLN 119	*	H	A:SER 43
134	H	A:LYS 119	*	H	A:ASP 120	*	H	A:LEU 44
135	H	A:GLY 120	*	H	A:LEU 121	*	H	A:SER 45
136	H	A:LEU 121	*	H	A:ILE 122	*	H	A:GLN 46
137	H	A:PRO 122	*	H	A:PRO 123	*	H	A:LYS 47
138	H	A:ARG 123	*	H	A:ILE 124	*	H	A:THR 48
139	H	A:VAL 124	*	H	A:LEU 125	*	H	A:VAL 49
140	H	A:LEU 125	*	H	A:HIS 126	*	H	A:LEU 50
141	H	A:THR 126	*	H	A:ASN 127	*	H	A:GLU 51
142	H	A:LEU 127	*	H	A:LEU 128	*	H	A:MET 52
143	H	A:GLN 128	*	H	A:GLU 129	*	H	A:LEU 53
144	H	A:TYR 129	*	H	A:THR 130	*	H	A:GLN 54
145		a:pro 130					h	a:thr 55
146		a:his 131					h	a:his 56
147		a:thr 132					h	a:leu 57

```
148 | |a:ala 133 | |           | |h|a:val  58 |
149 | |a:pro 134 | |           | | |a:ala  59 |
150 |h|a:cys 144 | |           | | |a:gly  60 |
151 |h|a:thr 145 | |           | |h|a:pro  61 |
152 |h|a:glu 146 | |           | |h|a:asp  62 |
153 |h|a:trp 147 | |           | |h|a:ala  63 |
154 |h|a:cys 148 | |           | |h|a:ser  64 |
155 |h|a:leu 149 | |           | |h|a:glu  65 |
156 |h|a:ser 150 | |           | |h|a:thr  66 |
157 | |a:his 151 | |           | |h|a:ile  67 |
158 | |a:phe 152 | |           | |h|a:arg  68 |
159 | |a:val 153 | |           | |h|a:arg  69 |
160 | |a:gly 154 | |           | |h|a:gln  70 |
161 | |a:ala 155 | |           | |h|a:val  71 |
162 |h|a:ser 156 | |           | |h|a:ala  72 |
163 |h|a:gly 157 | |           | |h|a:phe  73 |
164 |h|a:arg 158 | |           | |h|a:leu  74 |
165 |h|a:leu 159 | |           | |h|a:ile  75 |
166 |h|a:arg 160 | |           | |h|a:tyr  76 |
167 |h|a:ser 161 | |           | |h|a:asp  77 |
168 |h|a:glu 162 | |           | |h|a:phe  78 |
169 |h|a:val 163 | |           | |h|a:his  79 |
170 |h|a:arg 164 | |           | |h|a:arg  80 |
171 |h|a:asp 165 | |           | |h|a:leu  81 |
172 |h|a:ile 166 | |           | |h|a:ser  82 |
173 |h|a:leu 167 | |           | |h|a:cys  83 |
174 |h|a:thr 168 | |           | |h|a:asn  84 |
175 | |a:thr 169 | |           | |h|a:cys  85 |
176 | |a:his 170 | |           | |h|a:asp  86 |
177 | |a:asn 171 | |           | |h|a:lys  87 |
178 | |a:gly 172 | |           | | |a:cys  88 |
179 | |a:thr 173 | |           | | |a:tyr  89 |
180 | |a:cys 174 | |           | | |a:gly  90 |
181 | |a:ala 175 | |           | | |a:asp  91 |
182 | |a:pro 176 | |           | | |a:cys  92 |
183 |h|a:ser 177 | |           | | |a:asn  93 |
184 |h|a:phe 178 | |           | | |a:ala  94 |
185 |h|a:glu 179 | |           | |h|a:thr  95 |
186 |h|a:trp 180 | |           | |h|a:thr  96 |
187 |h|a:met 181 | |           | |h|a:thr  97 |
188 |h|a:ala 182 | |           | |h|a:gly  98 |
189 |h|a:ser 183 | |           | |h|a:arg  99 |
190 |h|a:val 184 | |           | |h|a:phe 100 |
191 |h|a:val 185 | |           | |h|a:lys 101 |
192 |h|a:lys 186 | |           | |h|a:val 102 |
193 |h|a:lys 187 | |           | |h|a:val 103 |
194 |h|a:phe 188 | |           | |h|a:asp 104 |
195 |h|a:phe 189 | |           | |h|a:arg 105 |
196 |h|a:leu 190 | |           | |h|a:val 106 |
197 | |a:val 191 | |           | |h|a:leu 107 |
```

```
198 | |a:glu 192 | |            | |h|a:asn 108 |
199 | |a:thr 193 | |            | |h|a:asp 109 |
200 | |a:val 194 | |            | |h|a:his 110 |
201 | |a:ile 195 | |            | |h|a:ile 111 |
202 | |a:tyr 196 | |            | |h|a:glu 112 |
203 | |a:glu 197 | |            | |h|a:phe 113 |
204 | |a:asp 198 | |            | |h|a:gly 114 |
205 | |a:phe 199 | |            | |h|a:ile 115 |
206 | |a:gln 200 | |            | |h|a:met 116 |
207 |h|a:asp 201 | |            | |h|a:arg 117 |
208 |h|a:thr 202 | |            | |h|a:arg 118 |
209 |h|a:asp 203 | |            | |h|a:gln 119 |
210 |h|a:phe 204 | |            | |h|a:asp 120 |
211 |h|a:asn 205 | |            | |h|a:leu 121 |
212 |h|a:val 206 | |            | |h|a:ile 122 |
213 |h|a:gln 207 | |            | |h|a:pro 123 |
214 |h|a:leu 208 | |            | |h|a:ile 124 |
215 |h|a:asn 209 | |            | |h|a:leu 125 |
216 |h|a:leu 210 | |            | |h|a:his 126 |
217 |h|a:cys 211 | |            | |h|a:asn 127 |
218 |h|a:phe 212 | |            | |h|a:leu 128 |
219 |h|a:phe 213 | |            | |h|a:glu 129 |
220 |h|a:trp 214 | |            | |h|a:thr 130 |
221 |h|a:thr 215 | |            | |            |
222 |h|a:ala 216 | |            | |            |
223 |h|a:val 217 | |            | |            |
224 |h|a:val 218 | |            | |            |
225 |h|a:gln 219 | |            | |            |
226 |h|a:met 220 | |            | |            |
227 |h|a:tyr 221 | |            | |            |
228 |h|a:gln 222 | |            | |            |
229 |h|a:arg 223 | |            | |            |
230 |h|a:cys 224 | |            | |            |
231 |h|a:ile 225 | |            | |            |
232 |h|a:tyr 226 | |            | |            |
233 |h|a:glu 227 | |            | |            |
234 |h|a:gln 228 | |            | |            |
235 |h|a:lys 229 | |            | |            |
236 |h|a:leu 230 | |            | |            |
237 |h|a:val 231 | |            | |            |
238 |h|a:his 232 | |            | |            |
239 |h|a:ile 233 | |            | |            |
240 |h|a:ile 234 | |            | |            |
241 |h|a:ser 235 | |            | |            |
242 |h|a:thr 236 | |            | |            |
243 |h|a:ser 237 | |            | |            |
244 |h|a:leu 238 | |            | |            |
245 |h|a:thr 239 | |            | |            |
246 |h|a:leu 240 | |            | |            |
247 |h|a:leu 241 | |            | |            |
```

```
248 |h|a:lys 242 | |            | |            |
249 |h|a:ser 243 | |            | |            |
250 |h|a:thr 244 | |            | |            |
251 |h|a:ala 245 | |            | |            |
252 |h|a:arg 246 | |            | |            |
253 |h|a:ser 247 | |            | |            |
254 |h|a:phe 248 | |            | |            |
255 |h|a:phe 249 | |            | |            |
256 |h|a:ala 250 | |            | |            |
257 |h|a:trp 251 | |            | |            |
258 |h|a:tyr 252 | |            | |            |
259 |h|a:asp 253 | |            | |            |
260 |h|a:leu 254 | |            | |            |
261 |h|a:tyr 255 | |            | |            |
262 |h|a:arg 256 | |            | |            |
263 | |a:pro 257 | |            | |            |
264 | |a:asn 258 | |            | |            |
265 | |a:leu 259 | |            | |            |
266 | |a:gly 260 | |            | |            |
267 |h|a:ser 261 | |            | |            |
268 |h|a:ala 262 | |            | |            |
269 |h|a:ala 263 | |            | |            |
270 |h|a:leu 264 | |            | |            |
271 |h|a:val 265 | |            | |            |
272 |h|a:lys 266 | |            | |            |
273 |h|a:tyr 267 | |            | |            |
274 |h|a:thr 268 | |            | |            |
275 |h|a:glu 269 | |            | |            |
276 |h|a:his 270 | |            | |            |
277 |h|a:leu 271 | |            | |            |
278 |h|a:ile 272 | |            | |            |
279 |h|a:arg 273 | |            | |            |
280 |h|a:ala 274 | |            | |            |
281 | |a:leu 275 | |            | |            |
282 | |a:thr 276 | |            | |            |
283 | |a:pro 277 | |            | |            |
284 | |a:asp 278 | |            | |            |
285 | |a:cys 279 | |            | |            |
286 |h|a:ser 280 | |            | |            |
287 |h|a:asp 281 | |            | |            |
288 |h|a:val 282 | |            | |            |
289 |h|a:glu 283 | |            | |            |
290 |h|a:leu 284 | |            | |            |
291 |h|a:gly 285 | |            | |            |
292 |h|a:glu 286 | |            | |            |
293 |h|a:leu 287 | |            | |            |
294 |h|a:cys 288 | |            | |            |
295 |h|a:ser 289 | |            | |            |
296 |h|a:his 290 | |            | |            |
297 |h|a:leu 291 | |            | |            |
```

```
 298 |h|a:his 292 | |            | |            |
 299 |h|a:his 293 | |            | |            |
 300 |h|a:cys 294 | |            | |            |
 301 |h|a:lys 295 | |            | |            |
 302 |h|a:his 296 | |            | |            |
 303 |h|a:ala 297 | |            | |            |
 304 |h|a:leu 298 | |            | |            |
 305 |h|a:phe 299 | |            | |            |
 306 | |a:ser 300 | |            | |            |
-----+-----------+-+------------+-+------------+
```
