# Supplementary material for: Poorly Conserved P15 Proteins of Cileviruses Retain Elements of Common Ancestry and Putative Functionality: A Theoretical Assessment on the Evolution of Cilevirus Genomes
Source: Front Plant Sci. 2021 Nov 5;12:771983. doi: 10.3389/fpls.2021.771983 (PMC8602818; doi:10.3389/fpls.2021.771983)
Supplement: Supplementary file 2 [file Data_Sheet_2.docx]

**Supplementary Table S1**. Description of the dataset of ORF *p15* used in this study. Those sequences involved in the 3D structure prediction are in-depth described in section A.

| **Virus** | **Isolate** | **GenBank or RefSeq accession number** | | **Protein**  **​isoelectric point/ molecular weight** | **Plant host** | **Collection date** | **Reference** |
| --- | --- | --- | --- | --- | --- | --- | --- |
|  |  | **ORF** | **Protein** |  |  |  |  |
| CiLV-C | SJP01 | KP336747.1 | AKJ79135.1 | 5.15 / 15,191.24 | *Citrus sinensis* | 2011 | (Ramos-González et al., 2016) |
|  | Crd01 | NC008170.1 | ABC75823.1 | 5.15 / 15,191.24 | *Citrus sinensis* | 2006 | (Locali-Fabris et al., 2006) |
|  | Ar02 | KR093081.1 | ALF45382.1 | 5.47 / 15,153.26 | *Citrus sinensis* | 2012 | (Ramos-González et al., 2016) |
|  | Prb03 | MT554548.1 | QUM93157.1 | 5.15 / 15191.24 | *Citrus reticulata* | 2018 | (Chabi-Jesus et al., 2021) |
| CiLV-C2 | Co | NC_038849.1 | YP_009508072.1 | 5.68 / 15,093.18 | *Citrus sinensis* | 2011 | (Roy et al., 2013) |
|  | Co2 | MT304693.1 | QUF60861.1 | 5.13 / 15056.12 | *Citrus* sp. | 2008 | (Chabi-Jesus et al., 2021) |
|  | Fla | MG253804.1 | ATW76025.1 | 4.81 / 14,992.82 | *Hibiscus rosa-sinensis* | 2016 | (Roy et al., 2018) |
|  | Hw | KC626784.1 | AGM16553.1 | 4.83 / 15,004.87 | *Hibiscus* sp. | 2011 | (Melzer et al., 2013) |
| PfGSV | BSB1 | MK804174.1 | QFU28432.1 | 4.82 / 14,718.66 | *Passiflora* sp. | 2003 | (Ramos-González et al., 2020) |
|  | Snp1 | MK804172.1 | QFU28425.1 | 4.94 / 14,766.75 | *Passiflora* sp. | 2016 |  |
|  | BJL1 | MK804176.1 | QFU28439.1 | 4.91 / 14,916.84 | *Passiflora* sp. | 2008 |  |
|  | Py1 | NA | NA | 5.08 / 14,952.94 | *Ligustrum* sp. | 2017 | unpublished |
|  | Cmp1 | NA | NA | 5.25 / 14,882.82 | *Thunbergia grandiflora* | 2017 |  |

Section A: Isolates involved in the 3D structure prediction.

NA: not yet available.

Section B: Other CiLV-C isolates

| **CiLV-C isolate** | **GenBank or RefSeq accession number** | | **Reference** | **CiLV-C isolate** | **GenBank or RefSeq accession number** | | **Reference** |
| --- | --- | --- | --- | --- | --- | --- | --- |
|  | **ORF** | **Protein** |  |  | **ORF** | **Protein** |  |
| Py1 | MT304692.1 | QUF60860.1 | (Ramos-González et al., 2016) | SJP_03 | KR093167 | ALF45440 | (Ramos-González et al., 2016) |
| Cmp1 | MT304691.1 | QUF60859.1 |  | SDM_01 | KT253469 | ALP83394 |  |
| AMP_01 | KR093078 | ALF45379 |  | SDM_02 | KT253470 | ALP83395 |  |
| AJU_01 | KR093079 | ALF45380 |  | SDM_03 | KT253471 | ALP83396 |  |
| ARA_01 | KR093080 | ALF45381 |  | SDM_04 | KT253472 | ALP83397 |  |
| BEL_01 | KR136415 | ALI31822 |  | SDM_05 | KT253473 | ALP83398 |  |
| BRM_02 | KR093084 | ALF45385 |  | SDM_06 | KT253474 | ALP83399 |  |
| BSB_01 | KR093085 | ALF45386 |  | TNG_01 | KR093102 | ALF45403 |  |
| CSB_01 | KR093086 | ALF45387 |  | TTI_01 | KR093103 | ALF45404 |  |
| CLN_01 | KR093082 | ALF45383 |  | TRN_01 | KR093104 | ALF45405 |  |
| CGZ_01 | KR093087 | ALF45388 |  | PA_01 | DQ388513 | ABG33780 | Guerra et al.,  (unpublished) |
| CCH_01 | KR093088 | ALF45389 |  | Jac01 | MT554544 | QUM93141 | (Chabi-Jesus et al., 2021) |
| CRD_02 | KR093089 | ALF45390 |  | Prb04 | MT554549 | QUM93161 |  |
| CSM_01 | KR093083 | ALF45384 |  | Urg01 | MT554552 | QUM93173 |  |
| GYN_01 | KR093090 | ALF45391 |  | AR06 | MT554542 | QUM93133 |  |
| LSP_01 | KR093091 | ALF45392 |  | Asu02 | MT554546 | QUM93149 |  |
| LDB_01 | KR093092 | ALF45393 |  | Lim01 | MT554545 | QUM93145 |  |
| MAO_01 | KR093093 | ALF45394 |  | SPa11 | MT554551 | QUM93169 |  |
| MGF_01 | KR093094 | ALF45395 |  | Jbt02 | MT554543 | QUM93137 |  |
| MRN_01 | KR136416 | ALI31823 |  | AR04 | MT554540 | QUM93125 |  |
| PMW_01 | KR093105 | ALF45406 |  | Prb02 | MT554547 | QUM93153 |  |
| PRB_01 | KR093095 | ALF45396 |  | AR05 | MT554542 | QUM93133 |  |
| PNT_01 | KR093096 | ALF45397 |  | Itb01 | MT304683 | QUF60851 |  |
| PRT_01 | KR093097 | ALF45398 |  | SdM15 | MW574408 | NA |  |
| ITU_01 | KR093099 | ALF45400 |  | Vtr01 | MT554553 | QUM93177 |  |
| SAP_01 | KR093098 | ALF45399 |  | CaP01 | MW574406 | NA |  |
| JBT_01 | DQ157465 | ALF45381 |  | Jmr01 | MW574407 | NA |  |
| SNG_01 | KR093100 | ALF45401 |  | Lim09 | MW574409 | NA |  |
| SJP_02 | KR093101 | ALF45402 |  | SAP03 | MW574410 | NA |  |

NA: not yet available.

**Supplementary Table S2**. Effective Number of codons (ENc) by ORF across cileviruses. Values were calculated using COUSIN bioinformatics tool (http://cousin.ird.fr/index.php). Values lower than 50 are underlined, and that under 40 is highlighted in boldface.

| **ORF** | **CiLV-C isolates** | | | **CiLV-C2 isolates** | | **PfGSV isolates** | | **Mean** | **SD**^1^ |
| --- | --- | --- | --- | --- | --- | --- | --- | --- | --- |
|  | **CRD** | **SJP** | **ASU** | **Co** | **Hw** | **Snp1** | **BJL** |  |  |
| *RdRp* | 51.075 | 50.867 | 48.755 | 52.223 | 51.255 | 50.099 | 50.603 | 50,697 | 1,076 |
| *p29* | 56.709 | 54.078 | 50.792 | 52.549 | 55.11 | 52.332 | 53.247 | 53,545 | 1,953 |
| *p15* | 53.848 | 53.707 | 52.731 | 44.419 | **35.722** | 47.727 | 55.567 | 49,103 | 7,089 |
| *p61* | 53.563 | 52.025 | 48.198 | 52.002 | 53.296 | 54.021 | 51.418 | 52,075 | 1,956 |
| *p32* | 54.693 | 48.596 | 53.109 | 47.848 | 54.64 | 46.189 | 46.437 | 50,216 | 3,802 |
| *p24* | 48.309 | 50.673 | 50.181 | 58.398 | 59.868 | 46.085 | 49.538 | 51,865 | 5,203 |
| **Mean** | 53,033 | 51,658 | 50,628 | 51,240 | 51,649 | 49,409 | 51,135 |  |  |
| **SD** | 2,945 | 2,057 | 2,011 | 4,744 | 8,307 | 3,303 | 3,133 |  |  |

^1^SD: standard deviation.

**Supplementary Table S3**. Relative synonymous codon usage (RSCU) across cilevirus ORFs calculated using the CAIcal server (https://ppuigbo.me/programs/CAIcal/). RSCU value > 1 means a codon with a positive codon usage bias, whereas RSCU values <1 indicate a negative codon usage bias. When the RSCU value is equal to 1.0, the codon is chosen equally and randomly.

| Virus/ORF | Codons | | | | | | | | | | | | | | | | | | | | | | | | | | | | | | | | | | | | | | | | | | | | | | | | | | | | | | | | | | |
| --- | --- | --- | --- | --- | --- | --- | --- | --- | --- | --- | --- | --- | --- | --- | --- | --- | --- | --- | --- | --- | --- | --- | --- | --- | --- | --- | --- | --- | --- | --- | --- | --- | --- | --- | --- | --- | --- | --- | --- | --- | --- | --- | --- | --- | --- | --- | --- | --- | --- | --- | --- | --- | --- | --- | --- | --- | --- | --- | --- |
|  | **TTT** | **TTC** | **TTA** | **TTG** | **CTT** | **CTC** | **CTA** | **CTG** | **ATT** | **ATC** | **ATA** | **GTT** | **GTC** | **GTA** | **GTG** | **TCT** | **TCC** | **TCA** | **TCG** | **AGT** | **AGC** | **CCT** | **CCC** | **CCA** | **CCG** | **ACT** | **ACC** | **ACA** | **ACG** | **GCT** | **GCC** | **GCA** | **GCG** | **TAT** | **TAC** | **CAT** | **CAC** | **CAA** | **CAG** | **AAT** | **AAC** | **AAA** | **AAG** | **GAT** | **GAC** | **GAA** | **GAG** | **TGT** | **TGC** | **CGT** | **CGC** | **CGA** | **CGG** | **AGA** | **AGG** | **GGT** | **GGC** | **GGA** | **GGG** |
| CiLV-C_CRD/RdRp | 1.333 | 0.667 | 0.905 | 1.681 | 1.681 | 0.491 | 0.543 | 0.698 | 1.589 | 0.834 | 0.576 | 1.639 | 1.004 | 0.652 | 0.705 | 1.768 | 0.696 | 1.043 | 0.725 | 1.130 | 0.638 | 1.882 | 0.565 | 1.035 | 0.518 | 1.373 | 1.015 | 1.045 | 0.567 | 1.965 | 0.456 | 0.667 | 0.912 | 1.181 | 0.819 | 1.324 | 0.676 | 0.750 | 1.250 | 1.293 | 0.707 | 0.624 | 1.376 | 1.196 | 0.804 | 0.773 | 1.227 | 1.000 | 1.000 | 2.026 | 0.795 | 0.278 | 0.278 | 0.874 | 1.748 | 2.542 | 0.542 | 0.475 | 0.441 |
| CiLV-C_CRD/p29 | 0.500 | 1.500 | 1.263 | 0.947 | 1.263 | 0.316 | 0.316 | 1.895 | 1.286 | 0.643 | 1.071 | 1.600 | 0.800 | 0.800 | 0.800 | 1.364 | 1.091 | 0.818 | 0.545 | 1.636 | 0.545 | 2.095 | 0.762 | 0.571 | 0.571 | 2.286 | 0.571 | 1.143 | 0.000 | 1.440 | 0.960 | 0.320 | 1.280 | 1.800 | 0.200 | 0.000 | 2.000 | 0.818 | 1.182 | 1.250 | 0.750 | 0.385 | 1.615 | 0.933 | 1.067 | 0.353 | 1.647 | 0.000 | 2.000 | 0.857 | 1.714 | 1.714 | 0.857 | 0.857 | 0.000 | 1.818 | 1.091 | 0.000 | 1.091 |
| CiLV-C2_Co/RdRp | 1.400 | 0.600 | 1.082 | 1.992 | 1.156 | 0.467 | 0.615 | 0.689 | 1.579 | 0.730 | 0.691 | 2.029 | 0.722 | 0.663 | 0.585 | 1.507 | 0.725 | 1.275 | 0.754 | 1.362 | 0.377 | 1.349 | 0.530 | 1.301 | 0.819 | 1.395 | 0.775 | 1.209 | 0.620 | 1.457 | 0.744 | 1.364 | 0.434 | 1.355 | 0.645 | 1.507 | 0.493 | 0.955 | 1.045 | 1.053 | 0.947 | 0.774 | 1.226 | 1.326 | 0.674 | 1.085 | 0.915 | 1.471 | 0.529 | 2.058 | 0.438 | 0.526 | 0.438 | 1.358 | 1.182 | 2.034 | 0.448 | 0.931 | 0.586 |
| CiLV-C2_Co/p29 | 1.600 | 0.400 | 1.200 | 1.500 | 2.100 | 0.300 | 0.300 | 0.600 | 2.200 | 0.600 | 0.200 | 2.087 | 1.043 | 0.348 | 0.522 | 1.579 | 0.947 | 0.632 | 0.947 | 1.579 | 0.316 | 1.697 | 0.485 | 1.091 | 0.727 | 2.286 | 0.571 | 0.000 | 1.143 | 1.667 | 1.000 | 0.833 | 0.500 | 1.000 | 1.000 | 0.500 | 1.500 | 1.222 | 0.778 | 0.750 | 1.250 | 0.545 | 1.455 | 1.333 | 0.667 | 0.714 | 1.286 | 1.500 | 0.500 | 2.824 | 0.706 | 0.353 | 0.000 | 1.412 | 0.706 | 1.867 | 0.267 | 1.067 | 0.800 |
| CiLV-C_SJP/RdRp | 1.474 | 0.526 | 1.190 | 1.570 | 1.722 | 0.506 | 0.532 | 0.481 | 1.355 | 0.910 | 0.735 | 1.780 | 0.969 | 0.511 | 0.740 | 1.861 | 0.722 | 1.250 | 0.389 | 1.361 | 0.417 | 1.789 | 0.474 | 1.158 | 0.579 | 1.692 | 0.800 | 1.015 | 0.492 | 2.085 | 0.479 | 0.991 | 0.444 | 1.410 | 0.590 | 1.417 | 0.583 | 0.800 | 1.200 | 1.241 | 0.759 | 0.649 | 1.351 | 1.322 | 0.678 | 0.782 | 1.218 | 1.235 | 0.765 | 1.686 | 0.902 | 0.431 | 0.314 | 1.373 | 1.294 | 2.276 | 0.690 | 0.517 | 0.517 |
| CiLV-C_SJP/p29 | 0.889 | 1.111 | 0.333 | 1.667 | 1.333 | 0.667 | 0.333 | 1.667 | 1.071 | 0.857 | 1.071 | 2.105 | 0.632 | 0.421 | 0.842 | 1.714 | 0.857 | 1.143 | 0.286 | 1.714 | 0.286 | 2.000 | 0.800 | 0.600 | 0.600 | 2.000 | 1.000 | 1.000 | 0.000 | 2.000 | 0.500 | 1.000 | 0.500 | 1.600 | 0.400 | 1.000 | 1.000 | 0.917 | 1.083 | 1.200 | 0.800 | 0.667 | 1.333 | 1.600 | 0.400 | 0.875 | 1.125 | 0.000 | 2.000 | 2.571 | 0.000 | 1.714 | 0.000 | 0.857 | 0.857 | 2.000 | 0.333 | 0.333 | 1.333 |
| CiLV-C_ASU/RdRp | 1.429 | 0.571 | 1.256 | 1.641 | 1.513 | 0.564 | 0.538 | 0.487 | 1.519 | 0.769 | 0.712 | 1.982 | 0.793 | 0.595 | 0.631 | 1.909 | 0.600 | 1.173 | 0.545 | 1.309 | 0.464 | 1.671 | 0.608 | 1.316 | 0.405 | 1.714 | 0.730 | 1.111 | 0.444 | 1.947 | 0.460 | 1.097 | 0.496 | 1.333 | 0.667 | 1.500 | 0.500 | 1.034 | 0.966 | 1.368 | 0.632 | 0.653 | 1.347 | 1.257 | 0.743 | 0.833 | 1.167 | 1.391 | 0.609 | 2.092 | 0.592 | 0.197 | 0.276 | 1.500 | 1.342 | 2.780 | 0.373 | 0.373 | 0.475 |
| CiLV-C_ASU/p29 | 1.111 | 0.889 | 0.667 | 1.333 | 2.333 | 0.000 | 0.333 | 1.333 | 1.412 | 0.529 | 1.059 | 1.684 | 1.053 | 0.632 | 0.632 | 2.000 | 1.143 | 0.571 | 0.286 | 1.714 | 0.286 | 1.636 | 0.545 | 0.727 | 1.091 | 1.500 | 0.500 | 2.000 | 0.000 | 2.364 | 0.545 | 0.727 | 0.364 | 1.600 | 0.400 | 0.500 | 1.500 | 0.727 | 1.273 | 1.111 | 0.889 | 0.320 | 1.680 | 1.714 | 0.286 | 1.111 | 0.889 | 0.000 | 2.000 | 1.500 | 0.750 | 2.250 | 0.000 | 0.750 | 0.750 | 2.545 | 0.727 | 0.000 | 0.727 |
| CiLV-C2_Hw/RdRp | 1.430 | 0.570 | 1.084 | 1.866 | 1.160 | 0.655 | 0.605 | 0.630 | 1.752 | 0.624 | 0.624 | 1.943 | 0.629 | 0.781 | 0.648 | 1.629 | 0.686 | 1.171 | 0.657 | 1.286 | 0.571 | 1.721 | 0.372 | 1.349 | 0.558 | 1.659 | 0.859 | 1.037 | 0.444 | 1.632 | 0.576 | 1.152 | 0.640 | 1.419 | 0.581 | 1.314 | 0.686 | 1.143 | 0.857 | 1.219 | 0.781 | 0.723 | 1.277 | 1.444 | 0.556 | 1.158 | 0.842 | 1.449 | 0.551 | 1.787 | 0.596 | 0.638 | 0.340 | 1.702 | 0.936 | 2.230 | 0.460 | 0.991 | 0.319 |
| CiLV-C2_Hw/p29 | 1.600 | 0.400 | 2.100 | 0.900 | 1.500 | 0.300 | 0.300 | 0.900 | 1.125 | 1.312 | 0.562 | 1.400 | 1.000 | 1.000 | 0.600 | 1.333 | 0.667 | 0.667 | 1.333 | 2.000 | 0.000 | 1.529 | 0.588 | 1.294 | 0.588 | 1.778 | 0.889 | 0.889 | 0.444 | 2.087 | 0.348 | 1.217 | 0.348 | 0.909 | 1.091 | 1.000 | 1.000 | 0.941 | 1.059 | 0.800 | 1.200 | 0.667 | 1.333 | 1.333 | 0.667 | 1.067 | 0.933 | 1.000 | 1.000 | 3.200 | 0.400 | 0.400 | 0.000 | 1.600 | 0.400 | 2.133 | 0.267 | 0.800 | 0.800 |
| PfGSV_Snp1/RdRp | 1.409 | 0.591 | 1.377 | 1.918 | 1.254 | 0.270 | 0.664 | 0.516 | 1.122 | 0.694 | 1.184 | 2.059 | 0.634 | 0.891 | 0.416 | 1.607 | 0.616 | 1.312 | 0.884 | 1.125 | 0.455 | 1.922 | 0.208 | 1.403 | 0.468 | 1.681 | 0.783 | 0.870 | 0.667 | 1.828 | 0.621 | 1.138 | 0.414 | 1.306 | 0.694 | 1.403 | 0.597 | 1.304 | 0.696 | 1.095 | 0.905 | 1.136 | 0.864 | 1.545 | 0.455 | 1.200 | 0.800 | 1.343 | 0.657 | 2.128 | 0.298 | 0.553 | 0.426 | 1.574 | 1.021 | 2.126 | 0.180 | 1.081 | 0.613 |
| PfGSV_Snp1/p29 | 1.143 | 0.857 | 2.182 | 1.091 | 1.909 | 0.000 | 0.273 | 0.545 | 1.800 | 0.900 | 0.300 | 1.565 | 0.174 | 1.391 | 0.870 | 2.769 | 0.462 | 0.923 | 0.923 | 0.923 | 0.000 | 1.333 | 0.121 | 1.697 | 0.848 | 0.000 | 0.889 | 1.333 | 1.778 | 2.545 | 0.545 | 0.727 | 0.182 | 1.200 | 0.800 | 0.800 | 1.200 | 1.714 | 0.286 | 0.600 | 1.400 | 0.667 | 1.333 | 1.333 | 0.667 | 1.286 | 0.714 | 1.000 | 1.000 | 1.826 | 0.261 | 0.783 | 0.522 | 1.304 | 1.304 | 1.714 | 0.286 | 0.571 | 1.429 |
| PfGSV_BJL1/RdRp | 1.507 | 0.493 | 1.333 | 1.877 | 1.383 | 0.198 | 0.667 | 0.543 | 1.224 | 0.632 | 1.145 | 2.162 | 0.586 | 0.747 | 0.505 | 1.656 | 0.706 | 1.303 | 0.760 | 1.167 | 0.407 | 1.722 | 0.304 | 1.418 | 0.557 | 1.547 | 0.876 | 0.876 | 0.701 | 1.744 | 0.650 | 0.957 | 0.650 | 1.271 | 0.729 | 1.362 | 0.638 | 1.246 | 0.754 | 1.188 | 0.812 | 1.013 | 0.987 | 1.512 | 0.488 | 1.161 | 0.839 | 1.486 | 0.514 | 2.014 | 0.336 | 0.420 | 0.503 | 1.804 | 0.923 | 1.910 | 0.216 | 1.189 | 0.685 |
| PfGSV_BJL1/p29 | 1.143 | 0.857 | 1.636 | 1.364 | 1.909 | 0.000 | 0.545 | 0.545 | 1.500 | 1.200 | 0.300 | 1.565 | 0.348 | 1.043 | 1.043 | 2.308 | 0.923 | 0.923 | 0.923 | 0.923 | 0.000 | 1.333 | 0.121 | 1.697 | 0.848 | 0.000 | 0.889 | 1.333 | 1.778 | 2.727 | 0.364 | 0.727 | 0.182 | 1.200 | 0.800 | 0.800 | 1.200 | 1.857 | 0.143 | 0.800 | 1.200 | 0.333 | 1.667 | 1.200 | 0.800 | 1.286 | 0.714 | 1.500 | 0.500 | 1.826 | 0.261 | 0.783 | 0.522 | 1.304 | 1.304 | 1.714 | 0.286 | 0.857 | 1.143 |
| CiLV-C_CRD/p15 | 1.000 | 1.000 | 1.000 | 1.000 | 1.000 | 1.500 | 0.500 | 1.000 | 2.100 | 0.000 | 0.900 | 2.286 | 0.571 | 0.000 | 1.143 | 4.286 | 0.857 | 0.000 | 0.000 | 0.857 | 0.000 | 0.000 | 0.000 | 2.667 | 1.333 | 1.200 | 1.200 | 0.400 | 1.200 | 0.800 | 1.600 | 0.800 | 0.800 | 1.500 | 0.500 | 0.857 | 1.143 | 1.333 | 0.667 | 1.200 | 0.800 | 0.667 | 1.333 | 1.500 | 0.500 | 0.667 | 1.333 | 1.143 | 0.857 | 2.571 | 0.000 | 0.857 | 0.000 | 1.714 | 0.857 | 2.400 | 0.000 | 0.000 | 1.600 |
| CiLV-C_CRD/p61 | 1.385 | 0.615 | 1.132 | 2.038 | 1.132 | 0.679 | 0.453 | 0.566 | 1.425 | 0.900 | 0.675 | 2.211 | 0.632 | 0.526 | 0.632 | 1.600 | 0.933 | 0.533 | 0.667 | 1.733 | 0.533 | 1.412 | 0.235 | 1.412 | 0.941 | 0.923 | 1.077 | 0.923 | 1.077 | 2.400 | 0.600 | 0.600 | 0.400 | 1.415 | 0.585 | 1.375 | 0.625 | 1.000 | 1.000 | 1.429 | 0.571 | 0.741 | 1.259 | 1.400 | 0.600 | 0.917 | 1.083 | 1.565 | 0.435 | 1.200 | 0.900 | 0.300 | 0.300 | 1.200 | 2.100 | 1.730 | 0.541 | 0.757 | 0.973 |
| CiLV-C_CRD/p32 | 1.600 | 0.400 | 0.818 | 1.636 | 1.909 | 0.273 | 0.818 | 0.545 | 1.364 | 0.273 | 1.364 | 1.806 | 1.032 | 0.258 | 0.903 | 1.784 | 0.811 | 1.135 | 1.297 | 0.811 | 0.162 | 0.667 | 0.000 | 1.667 | 1.667 | 1.905 | 0.571 | 0.571 | 0.952 | 2.000 | 0.571 | 0.857 | 0.571 | 2.000 | 0.000 | 1.000 | 1.000 | 1.143 | 0.857 | 1.556 | 0.444 | 0.667 | 1.333 | 1.059 | 0.941 | 0.588 | 1.412 | 1.500 | 0.500 | 1.263 | 0.947 | 0.632 | 0.632 | 1.263 | 1.263 | 2.000 | 0.500 | 0.750 | 0.750 |
| CiLV-C_CRD/p24 | 1.222 | 0.778 | 0.333 | 2.000 | 2.000 | 0.667 | 0.667 | 0.333 | 1.143 | 0.571 | 1.286 | 2.000 | 0.600 | 0.400 | 1.000 | 1.800 | 0.600 | 1.200 | 0.600 | 1.800 | 0.000 | 1.500 | 0.500 | 1.500 | 0.500 | 2.222 | 1.333 | 0.000 | 0.444 | 2.286 | 0.381 | 0.571 | 0.762 | 1.714 | 0.286 | 1.333 | 0.667 | 1.500 | 0.500 | 0.667 | 1.333 | 0.727 | 1.273 | 1.143 | 0.857 | 1.333 | 0.667 | 1.000 | 1.000 | 0.000 | 0.857 | 0.000 | 0.429 | 0.857 | 3.857 | 2.000 | 1.333 | 0.000 | 0.667 |
| CiLV-C_SJP/p15 | 1.000 | 1.000 | 1.000 | 1.000 | 1.000 | 1.500 | 0.500 | 1.000 | 2.100 | 0.000 | 0.900 | 2.286 | 0.571 | 0.000 | 1.143 | 4.286 | 0.857 | 0.000 | 0.000 | 0.857 | 0.000 | 0.000 | 0.000 | 2.667 | 1.333 | 1.200 | 1.200 | 0.400 | 1.200 | 0.800 | 1.600 | 0.800 | 0.800 | 1.500 | 0.500 | 0.571 | 1.429 | 1.000 | 1.000 | 1.200 | 0.800 | 0.667 | 1.333 | 1.500 | 0.500 | 0.667 | 1.333 | 1.143 | 0.857 | 2.571 | 0.000 | 0.857 | 0.000 | 1.714 | 0.857 | 2.400 | 0.000 | 0.000 | 1.600 |
| CiLV-C_SJP/p61 | 1.487 | 0.513 | 1.889 | 1.111 | 1.222 | 0.444 | 0.667 | 0.667 | 1.463 | 0.732 | 0.805 | 1.500 | 0.750 | 0.500 | 1.250 | 1.650 | 1.500 | 0.900 | 0.150 | 1.650 | 0.150 | 1.500 | 0.000 | 2.000 | 0.500 | 1.333 | 0.444 | 0.889 | 1.333 | 2.095 | 0.571 | 0.381 | 0.952 | 1.422 | 0.578 | 1.625 | 0.375 | 0.909 | 1.091 | 1.294 | 0.706 | 0.571 | 1.429 | 1.379 | 0.621 | 0.966 | 1.034 | 1.565 | 0.435 | 1.143 | 1.429 | 0.286 | 0.286 | 0.857 | 2.000 | 1.939 | 0.364 | 1.091 | 0.606 |
| CiLV-C_SJP/p32 | 1.600 | 0.400 | 0.818 | 1.909 | 2.455 | 0.273 | 0.273 | 0.273 | 1.875 | 0.375 | 0.750 | 1.379 | 1.241 | 0.552 | 0.828 | 2.270 | 0.324 | 0.649 | 1.946 | 0.811 | 0.000 | 0.800 | 0.000 | 1.200 | 2.000 | 1.647 | 0.235 | 1.176 | 0.941 | 2.000 | 0.500 | 1.250 | 0.250 | 2.000 | 0.000 | 0.750 | 1.250 | 0.857 | 1.143 | 1.579 | 0.421 | 0.500 | 1.500 | 1.333 | 0.667 | 0.375 | 1.625 | 1.000 | 1.000 | 1.263 | 0.947 | 0.947 | 0.000 | 1.895 | 0.947 | 2.353 | 0.471 | 0.941 | 0.235 |
| CiLV-C_SJP/p24 | 1.556 | 0.444 | 0.333 | 1.333 | 2.333 | 0.333 | 1.000 | 0.667 | 0.818 | 0.955 | 1.227 | 2.105 | 0.421 | 0.632 | 0.842 | 2.727 | 0.000 | 1.091 | 0.545 | 1.091 | 0.545 | 2.222 | 0.000 | 1.778 | 0.000 | 2.000 | 1.600 | 0.000 | 0.400 | 1.474 | 0.632 | 1.053 | 0.842 | 1.429 | 0.571 | 1.667 | 0.333 | 1.143 | 0.857 | 1.167 | 0.833 | 0.615 | 1.385 | 1.250 | 0.750 | 1.000 | 1.000 | 1.000 | 1.000 | 0.000 | 1.000 | 0.500 | 0.000 | 2.000 | 2.500 | 1.333 | 1.333 | 0.667 | 0.667 |
| CiLV-C_ASU/p15 | 1.000 | 1.000 | 1.000 | 1.000 | 1.000 | 1.500 | 0.500 | 1.000 | 2.100 | 0.000 | 0.900 | 2.286 | 0.571 | 0.000 | 1.143 | 4.286 | 0.857 | 0.000 | 0.000 | 0.857 | 0.000 | 0.000 | 0.000 | 2.667 | 1.333 | 2.000 | 0.400 | 0.400 | 1.200 | 0.800 | 1.600 | 0.800 | 0.800 | 1.500 | 0.500 | 1.143 | 0.857 | 0.667 | 1.333 | 1.200 | 0.800 | 0.667 | 1.333 | 1.500 | 0.500 | 0.667 | 1.333 | 1.143 | 0.857 | 2.571 | 0.000 | 0.857 | 0.000 | 1.714 | 0.857 | 2.400 | 0.000 | 0.000 | 1.600 |
| CiLV-C_ASU/p61 | 1.421 | 0.579 | 1.474 | 2.105 | 1.158 | 0.526 | 0.632 | 0.105 | 1.622 | 0.649 | 0.730 | 1.730 | 0.973 | 0.324 | 0.973 | 1.773 | 0.955 | 1.773 | 0.000 | 1.227 | 0.273 | 2.250 | 0.000 | 1.250 | 0.500 | 1.419 | 0.774 | 1.161 | 0.645 | 1.750 | 0.500 | 1.000 | 0.750 | 1.545 | 0.455 | 1.571 | 0.429 | 1.400 | 0.600 | 1.176 | 0.824 | 0.500 | 1.500 | 1.724 | 0.276 | 1.241 | 0.759 | 1.652 | 0.348 | 1.263 | 0.632 | 0.316 | 0.316 | 0.947 | 2.526 | 2.424 | 0.121 | 1.091 | 0.364 |
| CiLV-C_ASU/p24 | 1.600 | 0.400 | 0.300 | 2.400 | 2.100 | 0.000 | 1.200 | 0.000 | 1.826 | 0.261 | 0.913 | 1.867 | 0.800 | 0.267 | 1.067 | 2.500 | 0.333 | 1.000 | 1.500 | 0.667 | 0.000 | 0.364 | 0.364 | 1.455 | 1.818 | 1.684 | 0.842 | 0.842 | 0.632 | 2.118 | 0.471 | 0.706 | 0.706 | 1.333 | 0.667 | 1.500 | 0.500 | 1.143 | 0.857 | 1.158 | 0.842 | 1.125 | 0.875 | 1.059 | 0.941 | 1.176 | 0.824 | 1.500 | 0.500 | 1.579 | 0.632 | 1.263 | 0.000 | 1.263 | 1.263 | 1.412 | 0.706 | 1.176 | 0.706 |
| CiLV-C_ASU/p33 | 1.667 | 0.333 | 1.000 | 1.667 | 2.000 | 0.333 | 1.000 | 0.000 | 0.714 | 1.000 | 1.286 | 2.222 | 0.444 | 0.889 | 0.444 | 1.200 | 0.600 | 1.200 | 1.200 | 1.800 | 0.000 | 1.500 | 0.000 | 2.500 | 0.000 | 2.000 | 1.600 | 0.000 | 0.400 | 2.000 | 0.000 | 0.800 | 1.200 | 1.429 | 0.571 | 1.333 | 0.667 | 0.750 | 1.250 | 0.833 | 1.167 | 0.615 | 1.385 | 1.500 | 0.500 | 1.111 | 0.889 | 1.000 | 1.000 | 0.500 | 1.000 | 0.000 | 0.000 | 2.000 | 2.500 | 2.000 | 0.667 | 0.667 | 0.667 |
| CiLV-C2_Co/p15 | 1.250 | 0.750 | 2.000 | 0.400 | 1.200 | 0.800 | 1.200 | 0.400 | 2.400 | 0.600 | 0.000 | 2.667 | 0.000 | 0.444 | 0.889 | 1.636 | 0.000 | 2.727 | 0.545 | 0.545 | 0.545 | 2.000 | 0.000 | 1.333 | 0.667 | 0.800 | 1.600 | 1.600 | 0.000 | 0.000 | 0.000 | 4.000 | 0.000 | 0.667 | 1.333 | 1.600 | 0.400 | 0.000 | 2.000 | 2.000 | 0.000 | 2.000 | 0.000 | 1.500 | 0.500 | 1.000 | 1.000 | 1.818 | 0.182 | 1.600 | 0.400 | 0.800 | 0.400 | 2.000 | 0.800 | 1.000 | 0.500 | 1.500 | 1.000 |
| CiLV-C2_Co/p61 | 1.412 | 0.588 | 1.964 | 1.200 | 1.091 | 0.545 | 0.545 | 0.655 | 1.333 | 0.444 | 1.222 | 2.049 | 1.073 | 0.585 | 0.293 | 1.778 | 0.778 | 1.444 | 0.667 | 0.778 | 0.556 | 1.143 | 0.571 | 1.714 | 0.571 | 1.455 | 0.970 | 1.091 | 0.485 | 2.000 | 0.889 | 0.667 | 0.444 | 1.086 | 0.914 | 1.714 | 0.286 | 2.000 | 0.000 | 1.385 | 0.615 | 0.833 | 1.167 | 1.278 | 0.722 | 1.652 | 0.348 | 1.586 | 0.414 | 2.100 | 0.900 | 0.900 | 0.600 | 1.500 | 0.000 | 1.600 | 0.457 | 1.029 | 0.914 |
| CiLV-C2_Co/p32 | 1.571 | 0.429 | 1.000 | 2.500 | 2.250 | 0.250 | 0.000 | 0.000 | 1.304 | 0.913 | 0.783 | 2.286 | 0.762 | 0.190 | 0.762 | 2.400 | 1.371 | 0.686 | 0.686 | 0.686 | 0.171 | 1.455 | 0.727 | 1.091 | 0.727 | 1.176 | 0.471 | 1.882 | 0.471 | 2.250 | 0.750 | 0.250 | 0.750 | 2.000 | 0.000 | 1.429 | 0.571 | 1.000 | 1.000 | 1.333 | 0.667 | 0.900 | 1.100 | 1.200 | 0.800 | 1.091 | 0.909 | 0.667 | 1.333 | 1.412 | 0.706 | 0.353 | 0.353 | 2.824 | 0.353 | 2.737 | 0.000 | 0.421 | 0.842 |
| CiLV-C2_Co/p24 | 1.125 | 0.875 | 1.333 | 1.778 | 0.889 | 0.667 | 0.667 | 0.667 | 0.923 | 1.154 | 0.923 | 2.364 | 0.545 | 0.727 | 0.364 | 1.000 | 1.500 | 2.000 | 1.500 | 0.000 | 0.000 | 1.333 | 0.444 | 1.333 | 0.889 | 1.714 | 1.143 | 0.571 | 0.571 | 2.545 | 0.727 | 0.727 | 0.000 | 1.250 | 0.750 | 1.333 | 0.667 | 1.333 | 0.667 | 0.200 | 1.800 | 1.200 | 0.800 | 1.000 | 1.000 | 1.000 | 1.000 | 1.333 | 0.667 | 1.000 | 1.500 | 0.000 | 1.000 | 1.000 | 1.500 | 2.222 | 1.333 | 0.000 | 0.444 |
| CiLV-C2_Hw/p15 | 1.000 | 1.000 | 1.286 | 1.714 | 2.143 | 0.429 | 0.429 | 0.000 | 3.000 | 0.000 | 0.000 | 3.111 | 0.000 | 0.444 | 0.444 | 1.333 | 0.000 | 2.667 | 0.667 | 0.667 | 0.667 | 2.667 | 0.667 | 0.667 | 0.000 | 2.000 | 0.000 | 2.000 | 0.000 | 2.000 | 0.000 | 2.000 | 0.000 | 2.000 | 0.000 | 2.000 | 0.000 | 2.000 | 0.000 | 1.333 | 0.667 | 0.000 | 0.000 | 1.500 | 0.500 | 1.333 | 0.667 | 1.333 | 0.667 | 2.727 | 0.000 | 0.545 | 0.000 | 2.727 | 0.000 | 1.500 | 0.500 | 2.000 | 0.000 |
| CiLV-C2_Hw/p61 | 1.176 | 0.824 | 2.036 | 1.286 | 1.179 | 0.643 | 0.214 | 0.643 | 1.071 | 0.857 | 1.071 | 2.286 | 0.571 | 0.229 | 0.914 | 1.560 | 0.960 | 1.440 | 0.480 | 1.320 | 0.240 | 1.000 | 1.000 | 1.500 | 0.500 | 1.556 | 0.778 | 0.889 | 0.778 | 2.333 | 0.500 | 1.000 | 0.167 | 1.143 | 0.857 | 1.667 | 0.333 | 1.333 | 0.667 | 1.273 | 0.727 | 0.857 | 1.143 | 1.500 | 0.500 | 1.263 | 0.737 | 1.310 | 0.690 | 2.211 | 0.316 | 0.632 | 0.632 | 1.895 | 0.316 | 1.556 | 0.778 | 1.000 | 0.667 |
| CiLV-C2_Hw/p32 | 1.333 | 0.667 | 1.043 | 1.826 | 2.087 | 0.261 | 0.261 | 0.522 | 1.304 | 0.913 | 0.783 | 1.647 | 0.706 | 0.471 | 1.176 | 2.769 | 0.769 | 0.923 | 0.615 | 0.308 | 0.615 | 2.000 | 0.000 | 1.200 | 0.800 | 1.474 | 0.421 | 0.842 | 1.263 | 1.750 | 1.000 | 1.250 | 0.000 | 0.667 | 1.333 | 1.429 | 0.571 | 1.000 | 1.000 | 1.077 | 0.923 | 0.667 | 1.333 | 1.385 | 0.615 | 1.100 | 0.900 | 1.333 | 0.667 | 1.714 | 0.286 | 0.857 | 0.571 | 2.000 | 0.571 | 2.105 | 0.000 | 1.053 | 0.842 |
| CiLV-C2_Hw/p24 | 1.467 | 0.533 | 1.556 | 1.333 | 0.889 | 0.667 | 0.222 | 1.333 | 0.750 | 0.750 | 1.500 | 1.565 | 0.870 | 0.696 | 0.870 | 1.091 | 1.091 | 2.727 | 0.545 | 0.545 | 0.000 | 2.000 | 0.400 | 0.400 | 1.200 | 1.500 | 1.500 | 0.500 | 0.500 | 1.818 | 0.727 | 0.727 | 0.727 | 1.111 | 0.889 | 1.000 | 1.000 | 1.500 | 0.500 | 0.667 | 1.333 | 0.889 | 1.111 | 1.111 | 0.889 | 0.800 | 1.200 | 0.667 | 1.333 | 0.923 | 0.923 | 0.923 | 0.000 | 1.385 | 1.846 | 2.222 | 0.444 | 1.333 | 0.000 |
| PfGSV_Snp1/p15 | 1.333 | 0.667 | 3.000 | 0.000 | 1.200 | 0.600 | 0.000 | 1.200 | 1.000 | 1.333 | 0.667 | 2.222 | 0.000 | 0.889 | 0.889 | 2.182 | 0.000 | 2.182 | 0.545 | 1.091 | 0.000 | 1.600 | 0.800 | 0.000 | 1.600 | 0.500 | 1.500 | 1.000 | 1.000 | 0.000 | 0.000 | 4.000 | 0.000 | 1.000 | 1.000 | 2.000 | 0.000 | 1.333 | 0.667 | 1.000 | 1.000 | 0.000 | 0.000 | 1.333 | 0.667 | 1.556 | 0.444 | 1.667 | 0.333 | 1.500 | 0.500 | 1.000 | 1.000 | 1.000 | 1.000 | 1.200 | 0.400 | 0.400 | 2.000 |
| PfGSV_Snp1/p61 | 1.227 | 0.773 | 1.448 | 2.276 | 1.241 | 0.310 | 0.517 | 0.207 | 1.111 | 0.889 | 1.000 | 1.818 | 0.909 | 0.818 | 0.455 | 0.818 | 0.955 | 1.500 | 0.409 | 1.500 | 0.818 | 1.000 | 0.600 | 1.400 | 1.000 | 1.600 | 1.029 | 1.029 | 0.343 | 1.895 | 0.842 | 0.842 | 0.421 | 1.143 | 0.857 | 1.538 | 0.462 | 1.333 | 0.667 | 1.185 | 0.815 | 0.960 | 1.040 | 1.600 | 0.400 | 1.474 | 0.526 | 1.172 | 0.828 | 0.632 | 0.632 | 0.000 | 1.263 | 1.895 | 1.579 | 2.051 | 0.103 | 1.026 | 0.821 |
| PfGSV_Snp1/p32 | 1.571 | 0.429 | 2.400 | 1.500 | 1.800 | 0.000 | 0.000 | 0.300 | 0.808 | 0.808 | 1.385 | 2.333 | 0.167 | 0.667 | 0.833 | 2.000 | 0.833 | 1.333 | 0.833 | 0.500 | 0.500 | 1.714 | 1.429 | 0.571 | 0.286 | 2.000 | 1.143 | 0.857 | 0.000 | 1.684 | 0.632 | 0.632 | 1.053 | 1.667 | 0.333 | 1.667 | 0.333 | 1.500 | 0.500 | 1.077 | 0.923 | 0.875 | 1.125 | 1.571 | 0.429 | 1.048 | 0.952 | 1.000 | 1.000 | 3.300 | 0.300 | 0.300 | 0.300 | 0.900 | 0.900 | 1.647 | 0.471 | 1.647 | 0.235 |
| PfGSV_Snp1/p24 | 1.625 | 0.375 | 1.154 | 1.615 | 1.846 | 0.231 | 0.462 | 0.692 | 1.250 | 0.250 | 1.500 | 2.182 | 0.727 | 0.727 | 0.364 | 3.600 | 0.600 | 1.800 | 0.000 | 0.000 | 0.000 | 0.800 | 0.400 | 1.600 | 1.200 | 0.500 | 1.000 | 2.500 | 0.000 | 2.800 | 0.400 | 0.800 | 0.000 | 1.750 | 0.250 | 2.000 | 0.000 | 1.000 | 1.000 | 0.857 | 1.143 | 0.833 | 1.167 | 1.250 | 0.750 | 0.857 | 1.143 | 1.333 | 0.667 | 1.091 | 1.636 | 0.545 | 0.545 | 2.182 | 0.000 | 2.182 | 0.364 | 1.455 | 0.000 |
| PfGSV_BJL1/p15 | 1.200 | 0.800 | 2.182 | 0.545 | 1.091 | 1.091 | 0.545 | 0.545 | 0.667 | 1.667 | 0.667 | 2.000 | 0.000 | 0.667 | 1.333 | 2.000 | 0.000 | 1.000 | 1.500 | 1.500 | 0.000 | 1.600 | 0.800 | 0.800 | 0.800 | 1.455 | 0.727 | 1.091 | 0.727 | 0.000 | 0.000 | 2.000 | 2.000 | 2.000 | 0.000 | 2.000 | 0.000 | 1.000 | 1.000 | 1.333 | 0.667 | 0.000 | 0.000 | 1.556 | 0.444 | 1.750 | 0.250 | 1.600 | 0.400 | 1.500 | 0.500 | 0.500 | 1.000 | 1.000 | 1.500 | 1.200 | 0.400 | 0.400 | 2.000 |
| PfGSV_BJL1/p61 | 1.378 | 0.622 | 1.759 | 1.966 | 1.241 | 0.310 | 0.517 | 0.207 | 1.286 | 0.857 | 0.857 | 2.261 | 0.522 | 0.609 | 0.609 | 1.250 | 0.625 | 1.500 | 0.375 | 1.375 | 0.875 | 1.263 | 0.421 | 1.053 | 1.263 | 1.576 | 0.970 | 0.970 | 0.485 | 2.105 | 0.842 | 0.632 | 0.421 | 1.120 | 0.880 | 1.538 | 0.462 | 1.667 | 0.333 | 1.304 | 0.696 | 1.333 | 0.667 | 1.611 | 0.389 | 1.579 | 0.421 | 1.241 | 0.759 | 1.200 | 0.600 | 0.300 | 0.600 | 2.400 | 0.900 | 2.000 | 0.400 | 1.200 | 0.400 |
| PfGSV_BJL1/p32 | 1.714 | 0.286 | 2.100 | 1.500 | 2.100 | 0.000 | 0.000 | 0.300 | 0.720 | 0.840 | 1.440 | 2.333 | 0.167 | 0.500 | 1.000 | 1.784 | 1.135 | 1.297 | 0.811 | 0.486 | 0.486 | 2.462 | 0.615 | 0.615 | 0.308 | 1.867 | 1.333 | 0.800 | 0.000 | 1.263 | 0.842 | 0.842 | 1.053 | 1.667 | 0.333 | 1.667 | 0.333 | 1.250 | 0.750 | 1.077 | 0.923 | 0.875 | 1.125 | 1.571 | 0.429 | 1.143 | 0.857 | 1.000 | 1.000 | 3.000 | 0.300 | 0.600 | 0.300 | 1.200 | 0.600 | 1.647 | 0.471 | 1.647 | 0.235 |
| PfGSV_BJL1/p24 | 1.500 | 0.500 | 1.615 | 1.615 | 1.615 | 0.231 | 0.231 | 0.692 | 1.000 | 0.500 | 1.500 | 2.364 | 0.545 | 0.727 | 0.364 | 3.000 | 1.200 | 1.800 | 0.000 | 0.000 | 0.000 | 1.200 | 0.000 | 1.600 | 1.200 | 0.500 | 1.000 | 2.500 | 0.000 | 2.800 | 0.400 | 0.800 | 0.000 | 1.750 | 0.250 | 1.333 | 0.667 | 1.000 | 1.000 | 1.143 | 0.857 | 1.167 | 0.833 | 1.250 | 0.750 | 0.571 | 1.429 | 1.333 | 0.667 | 1.091 | 1.636 | 0.545 | 0.545 | 2.182 | 0.000 | 1.818 | 0.727 | 1.455 | 0.000 |

**Supplementary Table S4**. Codon usage frequency of the cilevirus natural and experimental hosts. Citrus (*Citrus* x *sinensis*) (A), Passion fruit (*Passiflora* *edulis*) (B), Orchids (*Oncidium* spp.) (C), *Arabidopsis thaliana* (D), and common bean (*Phaseolus* *vulgaris*) (E).

1. Citrus (*Citrus x sinensis*). Values retrieved from Codon Usage Database.

UUU 26.3(503145) UCU 23.9(457980) UAU 17.2(330199) UGU 10.6(203586)

UUC 16.0(306903) UCC 10.6(202867) UAC 10.7(204118) UGC 8.2(156675)

UUA 13.9(267012) UCA 21.8(417852) UAA 0.8( 15055) UGA 1.0( 18837)

UUG 25.5(487760) UCG 6.3(120738) UAG 0.5( 10117) UGG 12.6(240508)

CUU 25.0(478899) CCU 18.4(352709) CAU 15.5(297250) CGU 7.2(137006)

CUC 11.5(219455) CCC 6.8(130591) CAC 7.9(151155) CGC 4.3( 81829)

CUA 9.2(176708) CCA 17.3(330496) CAA 20.1(385135) CGA 6.1(116013)

CUG 13.6(260831) CCG 5.3(101915) CAG 17.5(334395) CGG 4.9( 94154)

AUU 27.1(519539) ACU 17.8(340924) AAU 29.4(563782) AGU 16.0(305918)

AUC 12.6(242023) ACC 8.7(167169) AAC 15.7(299766) AGC 11.3(216885)

AUA 13.8(264299) ACA 15.9(305169) AAA 29.5(565672) AGA 16.9(324414)

AUG 23.8(455362) ACG 4.8( 91575) AAG 31.1(596047) AGG 13.1(250702)

GUU 27.3(523090) GCU 28.6(546798) GAU 37.9(726351) GGU 19.8(379865)

GUC 10.3(197615) GCC 11.6(221215) GAC 15.2(291984) GGC 11.8(225126)

GUA 10.6(202462) GCA 22.6(431944) GAA 35.1(673018) GGA 20.8(398389)

GUG 17.1(327594) GCG 5.3(100778) GAG 29.6(566004) GGG 12.3(235122)

1. Passion fruit (*Passiflora* *edulis*). Values calculated using countcodon v4.

UUU 20.1( 2020) UCU 16.5( 1662) UAU 13.8( 1390) UGU 17.4( 1751)

UUC 19.6( 1974) UCC 15.7( 1580) UAC 11.6( 1162) UGC 18.8( 1887)

UUA 12.1( 1217) UCA 19.0( 1915) UAA 10.1( 1016) UGA 23.7( 2383)

UUG 22.8( 2289) UCG 9.9( 998) UAG 10.7( 1078) UGG 25.1( 2526)

CUU 17.4( 1747) CCU 13.7( 1377) CAU 15.8( 1585) CGU 7.0( 700)

CUC 14.4( 1446) CCC 9.1( 918) CAC 12.5( 1253) CGC 5.4( 543)

CUA 10.8( 1084) CCA 18.0( 1810) CAA 22.1( 2223) CGA 11.7( 1182)

CUG 18.0( 1812) CCG 9.2( 928) CAG 21.9( 2204) CGG 9.8( 986)

AUU 17.7( 1785) ACU 12.7( 1273) AAU 16.1( 1615) AGU 14.8( 1493)

AUC 14.9( 1499) ACC 12.7( 1276) AAC 13.9( 1401) AGC 16.3( 1642)

AUA 11.9( 1198) ACA 16.1( 1623) AAA 21.0( 2112) AGA 25.9( 2605)

AUG 23.2( 2332) ACG 7.6( 766) AAG 22.7( 2283) AGG 19.3( 1938)

GUU 17.6( 1769) GCU 16.6( 1667) GAU 19.9( 2002) GGU 15.7( 1584)

GUC 11.5( 1160) GCC 12.3( 1237) GAC 11.4( 1146) GGC 13.5( 1363)

GUA 9.2( 929) GCA 17.7( 1785) GAA 23.7( 2386) GGA 22.9( 2307)

GUG 15.0( 1513) GCG 5.9( 594) GAG 22.3( 2240) GGG 14.2( 1430)

1. Orchids (*Oncidium* spp.). Values retrieved from Codon Usage Database.

UUU 27.3( 260) UCU 16.5( 157) UAU 19.3( 184) UGU 5.8( 55)

UUC 24.2( 230) UCC 11.0( 105) UAC 16.6( 158) UGC 9.4( 89)

UUA 14.0( 133) UCA 12.3( 117) UAA 0.4( 4) UGA 1.1( 10)

UUG 21.5( 205) UCG 6.0( 57) UAG 0.8( 8) UGG 14.7( 140)

CUU 26.9( 256) CCU 13.1( 125) CAU 13.1( 125) CGU 4.6( 44)

CUC 16.7( 159) CCC 6.8( 65) CAC 9.4( 89) CGC 4.6( 44)

CUA 8.0( 76) CCA 18.4( 175) CAA 13.7( 130) CGA 4.5( 43)

CUG 13.4( 127) CCG 8.0( 76) CAG 14.1( 134) CGG 4.9( 47)

AUU 28.1( 267) ACU 18.7( 178) AAU 29.1( 277) AGU 9.9( 94)

AUC 14.9( 142) ACC 12.0( 114) AAC 15.7( 149) AGC 11.7( 111)

AUA 17.9( 170) ACA 17.3( 165) AAA 26.8( 255) AGA 14.6( 139)

AUG 24.1( 229) ACG 4.2( 40) AAG 34.8( 331) AGG 17.2( 164)

GUU 23.1( 220) GCU 26.7( 254) GAU 37.9( 361) GGU 17.4( 166)

GUC 14.0( 133) GCC 15.9( 151) GAC 15.8( 150) GGC 17.2( 164)

GUA 7.9( 75) GCA 21.5( 205) GAA 28.4( 270) GGA 19.1( 182)

GUG 22.3( 212) GCG 9.3( 88) GAG 33.0( 314) GGG 12.2( 116)

1. *Arabidopsis thaliana.* Values retrieved from Codon Usage Database.

UUU 22.3(324240) UCU 25.6(371931) UAU 15.0(217876) UGU 10.9(158168)

UUC 19.9(288881) UCC 11.0(159579) UAC 13.1(190242) UGC 7.3(106514)

UUA 13.2(191655) UCA 19.1(276616) UAA 0.9( 12910) UGA 1.1( 15629)

UUG 21.3(309878) UCG 9.1(132184) UAG 0.5( 7442) UGG 12.4(179318)

CUU 24.5(354915) CCU 18.7(271153) CAU 14.2(206305) CGU 8.7(126349)

CUC 15.4(223146) CCC 5.2( 75263) CAC 8.5(122671) CGC 3.7( 54223)

CUA 10.2(147869) CCA 16.3(236254) CAA 19.9(289128) CGA 6.4( 92540)

CUG 10.2(147946) CCG 8.0(116583) CAG 15.4(223625) CGG 4.9( 70544)

AUU 21.8(315881) ACU 17.4(252946) AAU 23.3(337838) AGU 14.7(213090)

AUC 17.7(256873) ACC 9.8(142564) AAC 20.4(296234) AGC 11.5(166767)

AUA 13.3(192593) ACA 16.1(233332) AAA 31.3(454743) AGA 19.3(280404)

AUG 24.5(355750) ACG 7.4(106740) AAG 32.2(467906) AGG 11.0(159533)

GUU 27.0(392030) GCU 27.6(400630) GAU 37.3(541130) GGU 21.4(311213)

GUC 12.2(177223) GCC 9.7(140319) GAC 16.8(243463) GGC 8.9(128852)

GUA 10.3(149785) GCA 17.8(258968) GAA 35.4(514311) GGA 23.6(341875)

GUG 17.0(246151) GCG 8.4(121829) GAG 32.1(465496) GGG 10.2(147549)

1. Common bean *(Phaseolus vulgaris).* Values retrieved from Codon Usage Database.

UUU 22.6( 2979) UCU 21.5( 2843) UAU 15.6( 2064) UGU 7.9( 1047)

UUC 23.3( 3074) UCC 16.7( 2205) UAC 15.4( 2031) UGC 8.3( 1092)

UUA 9.7( 1279) UCA 15.8( 2088) UAA 0.7( 92) UGA 0.9( 115)

UUG 22.3( 2947) UCG 4.9( 649) UAG 0.7( 95) UGG 13.5( 1777)

CUU 23.6( 3113) CCU 17.7( 2333) CAU 12.8( 1696) CGU 7.1( 941)

CUC 18.2( 2409) CCC 10.7( 1417) CAC 10.7( 1418) CGC 5.5( 720)

CUA 9.4( 1244) CCA 17.2( 2273) CAA 18.7( 2472) CGA 4.2( 548)

CUG 13.3( 1760) CCG 4.1( 541) CAG 14.6( 1923) CGG 2.8( 372)

AUU 22.2( 2928) ACU 16.1( 2130) AAU 23.4( 3088) AGU 12.1( 1603)

AUC 18.7( 2470) ACC 17.0( 2244) AAC 27.1( 3579) AGC 11.8( 1556)

AUA 12.0( 1583) ACA 15.0( 1981) AAA 29.9( 3946) AGA 13.2( 1744)

AUG 21.3( 2807) ACG 5.8( 770) AAG 32.4( 4272) AGG 12.1( 1592)

GUU 23.6( 3117) GCU 24.7( 3266) GAU 32.6( 4305) GGU 22.7( 2997)

GUC 12.2( 1605) GCC 17.2( 2265) GAC 21.3( 2819) GGC 13.7( 1809)

GUA 7.6( 1007) GCA 19.8( 2610) GAA 31.9( 4207) GGA 23.2( 3057)

GUG 21.8( 2880) GCG 5.3( 696) GAG 29.3( 3867) GGG 12.8( 1691)

**Supplementary Table S5**. COUSIN values corresponding to each cilevirus ORF. Values were calculated using the codon usage of hosts described in Supplementary Table 4. COUSIN values higher than 1.5 in the *p15* column are underlined. The coefficient of variation of ORF *p15* is highlighted in boldface. Calculations were carried out using both natural and experimental host plants (A) and considering either natural (B) or experimental hosts (C).

1. Complete set of host plants.

| **Virus strain/host**^1^ | **COUSIN** | | | | | | | | |
| --- | --- | --- | --- | --- | --- | --- | --- | --- | --- |
|  | **Viral ORF** | | | | | | **basic statistical data**^2^ | | |
|  | *RdRp* | *p29* | *p15* | *p61* | *p32* | *p24* | Mean | SD | CV (%) |
| CiLV-C_CRD/At | 1.262 | 0.447 | 1.004 | 1.623 | 1.071 | 1.485 | 1.149 | 0.417 | 36.320 |
| CiLV-C_CRD/Cs | 0.795 | 0.238 | 0.849 | 1.203 | 0.916 | 1.314 | 0.886 | 0.378 | 42.663 |
| CiLV-C_CRD/On | 1.435 | 1.199 | 0.819 | 1.245 | 1.119 | 1.035 | 1.142 | 0.208 | 18.201 |
| CiLV-C_CRD/Pe | 1.192 | 0.208 | 0.805 | 1.402 | 0.839 | 1.485 | 0.989 | 0.474 | 47.967 |
| CiLV-C_CRD/Phv | 0.621 | 0.376 | 0.563 | 0.702 | 0.157 | 1.345 | 0.627 | 0.402 | 64.127 |
| CiLV-C_SJP/At | 1.425 | 1.014 | 0.815 | 1.646 | 1.321 | 1.569 | 1.298 | 0.324 | 24.961 |
| CiLV-C_SJP/Cs | 0.992 | 0.893 | 0.607 | 1.232 | 0.976 | 1.243 | 0.991 | 0.236 | 23.843 |
| CiLV-C_SJP/On | 1.548 | 1.348 | 0.868 | 1.51 | 1.586 | 1.333 | 1.366 | 0.265 | 19.422 |
| CiLV-C_SJP/Pe | 1.298 | 0.819 | 0.512 | 1.371 | 0.811 | 1.65 | 1.077 | 0.429 | 39.838 |
| CiLV-C_SJP/Phv | 0.628 | 1.03 | 0.354 | 0.662 | 0.36 | 0.708 | 0.624 | 0.251 | 40.306 |
| CiLV-C_Asu/At | 1.559 | 1.28 | 0.904 | 2.264 | 1.32 | 1.189 | 1.419 | 0.465 | 32.758 |
| CiLV-C_Asu/Cs | 1.195 | 0.924 | 0.622 | 1.833 | 0.937 | 1.043 | 1.092 | 0.409 | 37.417 |
| CiLV-C_Asu/On | 1.389 | 1.474 | 1.297 | 1.429 | 0.786 | 1.267 | 1.274 | 0.251 | 19.737 |
| CiLV-C_Asu/Pe | 1.496 | 1.297 | 0.606 | 2.219 | 1.169 | 1.064 | 1.309 | 0.536 | 40.971 |
| CiLV-C_Asu/Phv | 0.678 | 1.008 | 0.473 | 1.391 | 0.571 | 0.883 | 0.834 | 0.337 | 40.369 |
| CiLV-C2_Co/At | 1.558 | 0.997 | 1.514 | 1.731 | 1.876 | 0.683 | 1.393 | 0.458 | 32.898 |
| CiLV-C2_Co/Cs | 1.222 | 1.016 | 1.226 | 1.356 | 1.452 | 0.527 | 1.133 | 0.331 | 29.242 |
| CiLV-C2_Co/On | 1.135 | 0.797 | 0.821 | 0.796 | 1.149 | 0.309 | 0.835 | 0.306 | 36.679 |
| CiLV-C2_Co/Pe | 1.532 | 0.906 | 1.148 | 1.406 | 1.813 | 0.833 | 1.273 | 0.380 | 29.815 |
| CiLV-C2_Co/Phv | 0.92 | 0.865 | 0.3 | 0.498 | 1.188 | 0.565 | 0.723 | 0.325 | 45.040 |
| CiLV-C2_Hw/At | 1.871 | 0.734 | 2.171 | 1.663 | 1.545 | 0.868 | 1.475 | 0.566 | 38.331 |
| CiLV-C2_Hw/Cs | 1.436 | 0.697 | 2.001 | 1.194 | 1.229 | 0.885 | 1.240 | 0.456 | 36.781 |
| CiLV-C2_Hw/On | 1.07 | 0.703 | 1.022 | 0.977 | 1.103 | 0.695 | 0.928 | 0.183 | 19.683 |
| CiLV-C2_Hw/Pe | 1.949 | 0.417 | 1.842 | 1.546 | 1.526 | 1.026 | 1.384 | 0.572 | 41.354 |
| CiLV-C2_Hw/Phv | 1.02 | 0.689 | 1.274 | 0.652 | 1.033 | 0.836 | 0.917 | 0.237 | 25.810 |
| PfGSV_Snp1/At | 1.292 | 1.337 | 1.346 | 1.612 | 1.415 | 1.51 | 1.419 | 0.121 | 8.560 |
| PfGSV_Snp1/Pe | 1.2 | 1.236 | 0.776 | 1.493 | 1.343 | 1.569 | 1.270 | 0.281 | 22.128 |
| PfGSV_Snp1/On | 0.701 | 0.409 | -0.15 | 0.856 | 0.982 | 1.663 | 0.744 | 0.604 | 81.304 |
| PfGSV_Snp1/Cs | 0.972 | 1.118 | 1.153 | 1.173 | 1.264 | 1.478 | 1.193 | 0.169 | 14.162 |
| PfGSV_Snp1/Phv | 0.601 | 1.311 | 0.16 | 0.648 | 0.963 | 0.911 | 0.766 | 0.391 | 51.095 |
| PfGSV_BJL1/At | 1.485 | 1.368 | 1.448 | 1.536 | 1.498 | 1.105 | 1.407 | 0.158 | 11.259 |
| PfGSV_BJL1/Pe | 1.477 | 1.257 | 0.685 | 1.453 | 1.584 | 1.241 | 1.283 | 0.322 | 25.071 |
| PfGSV_BJL1/On | 0.796 | 0.484 | 0.09 | 0.942 | 0.898 | 1.506 | 0.786 | 0.476 | 60.510 |
| PfGSV_BJL1/Cs | 1.176 | 1.207 | 1.086 | 1.164 | 1.366 | 0.886 | 1.148 | 0.158 | 13.753 |
| PfGSV_BJL1/Phv | 0.704 | 1.244 | 0.21 | 0.543 | 1.109 | 0.287 | 0.683 | 0.424 | 62.022 |
| Mean | 1.189 | 0.924 | 0.892 | 1.285 | 1.151 | 1.086 |  |  |  |
| SD | 0.356 | 0.354 | 0.526 | 0.438 | 0.370 | 0.377 |  |  |  |
| CV (%) | 29.915 | 38.302 | **58.974** | 34.126 | 32.171 | 34.745 |  |  |  |

^1^At: *Arabidopsis thaliana*, Cs: *Citrus* x *sinensis*, Pe: *Passiflora edulis*, On: *Oncidium* sp., and Phv: *Phaseolus vulgaris*. ^2^SD: standard deviation, CV: coefficient of variation.

B. Natural host plants.

| **Virus strain/host**^1^ | **COUSIN** | | | | | | | | |
| --- | --- | --- | --- | --- | --- | --- | --- | --- | --- |
|  | **Viral ORF** | | | | | | **basic statistical data**^2^ | | |
|  | *RdRp* | *p29* | *p15* | *p61* | *p32* | *p24* | Mean | SD | CV (%) |
| CiLV-C_CRD/Cs | 0.795 | 0.238 | 0.849 | 1.203 | 0.916 | 1.314 | 0.886 | 0.378 | 42.663 |
| CiLV-C_CRD/On | 1.435 | 1.199 | 0.819 | 1.245 | 1.119 | 1.035 | 1.142 | 0.208 | 18.201 |
| CiLV-C_CRD/Pe | 1.192 | 0.208 | 0.805 | 1.402 | 0.839 | 1.485 | 0.989 | 0.474 | 47.967 |
| CiLV-C_SJP/Cs | 0.992 | 0.893 | 0.607 | 1.232 | 0.976 | 1.243 | 0.991 | 0.236 | 23.843 |
| CiLV-C_SJP/On | 1.548 | 1.348 | 0.868 | 1.51 | 1.586 | 1.333 | 1.366 | 0.265 | 19.422 |
| CiLV-C_SJP/Pe | 1.298 | 0.819 | 0.512 | 1.371 | 0.811 | 1.65 | 1.077 | 0.429 | 39.838 |
| CiLV-C_Asu/Cs | 1.195 | 0.924 | 0.622 | 1.833 | 0.937 | 1.043 | 1.092 | 0.409 | 37.417 |
| CiLV-C_Asu/On | 1.389 | 1.474 | 1.297 | 1.429 | 0.786 | 1.267 | 1.274 | 0.251 | 19.737 |
| CiLV-C_Asu/Pe | 1.496 | 1.297 | 0.606 | 2.219 | 1.169 | 1.064 | 1.309 | 0.536 | 40.971 |
| CiLV-C2_Co/Cs | 1.222 | 1.016 | 1.226 | 1.356 | 1.452 | 0.527 | 1.133 | 0.331 | 29.242 |
| CiLV-C2_Co/On | 1.135 | 0.797 | 0.821 | 0.796 | 1.149 | 0.309 | 0.835 | 0.306 | 36.679 |
| CiLV-C2_Co/Pe | 1.532 | 0.906 | 1.148 | 1.406 | 1.813 | 0.833 | 1.273 | 0.380 | 29.815 |
| CiLV-C2_Hw/Cs | 1.436 | 0.697 | 2.001 | 1.194 | 1.229 | 0.885 | 1.240 | 0.456 | 36.781 |
| CiLV-C2_Hw/On | 1.07 | 0.703 | 1.022 | 0.977 | 1.103 | 0.695 | 0.928 | 0.183 | 19.683 |
| CiLV-C2_Hw/Pe | 1.949 | 0.417 | 1.842 | 1.546 | 1.526 | 1.026 | 1.384 | 0.572 | 41.354 |
| PfGSV_Snp1/Pe | 1.2 | 1.236 | 0.776 | 1.493 | 1.343 | 1.569 | 1.270 | 0.281 | 22.128 |
| PfGSV_Snp1/On | 0.701 | 0.409 | -0.15 | 0.856 | 0.982 | 1.663 | 0.744 | 0.604 | 81.304 |
| PfGSV_Snp1/Cs | 0.972 | 1.118 | 1.153 | 1.173 | 1.264 | 1.478 | 1.193 | 0.169 | 14.162 |
| PfGSV_BJL1/Pe | 1.477 | 1.257 | 0.685 | 1.453 | 1.584 | 1.241 | 1.283 | 0.322 | 25.071 |
| PfGSV_BJL1/On | 0.796 | 0.484 | 0.09 | 0.942 | 0.898 | 1.506 | 0.786 | 0.476 | 60.510 |
| PfGSV_BJL1/Cs | 1.176 | 1.207 | 1.086 | 1.164 | 1.366 | 0.886 | 1.148 | 0.158 | 13.753 |
| Mean | 1.238 | 0.888 | 0.890 | 1.324 | 1.183 | 1.145 |  |  |  |
| SD | 0.298 | 0.376 | 0.489 | 0.322 | 0.292 | 0.369 |  |  |  |
| CV | 24.061 | 42.379 | **54.956** | 24.332 | 24.642 | 32.176 |  |  |  |

^1^Cs: *Citrus* x *sinensis*, Pe: *Passiflora edulis*, and On: *Oncidium* sp. ^2^SD: standard deviation, CV: coefficient of variation.

C. Experimental host plants.

| **Virus strain/host**^1^ | **COUSIN** | | | | | | | | |
| --- | --- | --- | --- | --- | --- | --- | --- | --- | --- |
|  | **Viral ORF** | | | | | | **basic statistical data**^2^ | | |
|  | *RdRp* | *p29* | *p15* | *p61* | *p32* | *p24* | Mean | SD | CV (%) |
| CiLV-C_CRD/At | 1.262 | 0.447 | 1.004 | 1.623 | 1.071 | 1.485 | 1.149 | 0.417 | 36.320 |
| CiLV-C_CRD/Phv | 0.621 | 0.376 | 0.563 | 0.702 | 0.157 | 1.345 | 0.627 | 0.402 | 64.127 |
| CiLV-C_SJP/At | 1.425 | 1.014 | 0.815 | 1.646 | 1.321 | 1.569 | 1.298 | 0.324 | 24.961 |
| CiLV-C_SJP/Phv | 0.628 | 1.03 | 0.354 | 0.662 | 0.36 | 0.708 | 0.624 | 0.251 | 40.306 |
| CiLV-C_Asu/At | 1.559 | 1.28 | 0.904 | 2.264 | 1.32 | 1.189 | 1.419 | 0.465 | 32.758 |
| CiLV-C_Asu/Phv | 0.678 | 1.008 | 0.473 | 1.391 | 0.571 | 0.883 | 0.834 | 0.337 | 40.369 |
| CiLV-C2_Co/At | 1.558 | 0.997 | 1.514 | 1.731 | 1.876 | 0.683 | 1.393 | 0.458 | 32.898 |
| CiLV-C2_Co/Phv | 0.92 | 0.865 | 0.3 | 0.498 | 1.188 | 0.565 | 0.723 | 0.325 | 45.040 |
| CiLV-C2_Hw/At | 1.871 | 0.734 | 2.171 | 1.663 | 1.545 | 0.868 | 1.475 | 0.566 | 38.331 |
| CiLV-C2_Hw/Phv | 1.02 | 0.689 | 1.274 | 0.652 | 1.033 | 0.836 | 0.917 | 0.237 | 25.810 |
| PfGSV_Snp1/At | 1.292 | 1.337 | 1.346 | 1.612 | 1.415 | 1.51 | 1.419 | 0.121 | 8.560 |
| PfGSV_Snp1/Phv | 0.601 | 1.311 | 0.16 | 0.648 | 0.963 | 0.911 | 0.766 | 0.391 | 51.095 |
| PfGSV_BJL1/At | 1.485 | 1.368 | 1.448 | 1.536 | 1.498 | 1.105 | 1.407 | 0.158 | 11.259 |
| PfGSV_BJL1/Phv | 0.704 | 1.244 | 0.21 | 0.543 | 1.109 | 0.287 | 0.683 | 0.424 | 62.022 |
| Mean | 1.116 | 0.979 | 0.895 | 1.227 | 1.102 | 0.996 |  |  |  |
| SD | 0.430 | 0.323 | 0.597 | 0.581 | 0.473 | 0.386 |  |  |  |
| CV | 38.526 | 33.044 | **66.627** | 47.339 | 42.889 | 38.725 |  |  |  |

^1^At: *Arabidopsis thaliana*, Phv: *Phaseolus vulgaris*. ^2^SD: standard deviation, CV: coefficient of variation.

**Supplementary Table S6**. RCDI/eRCDI ratio values corresponding to each cilevirus ORF. Ratio values were calculated using the codon usage tables of the hosts described in Supplementary Table 4. The lowest values in the *p15* column are underlined. The RCDI/eRCDI ratio mean value of ORF *p15* is highlighted in boldface. Calculations were carried out using both natural and experimental host plants (A) and considering either natural (B) or experimental hosts (C). Software settings were as follows: significance level and coverage in the estimation of an expected RCDI; confidence: 99%; population 95%; the method for estimating the expected RCDI value: Markov; and length of random sequences: 300.

1. Complete set of host plants.

| **Virus strain/host^1^** | **RCDI/eRCDI ratio** | | | | | | | | |
| --- | --- | --- | --- | --- | --- | --- | --- | --- | --- |
|  | **ORF** | | | | | | **basic statistical data^2^** | | |
|  | *RdRp* | *p29* | *p15* | *p61* | *p32* | *p24* | Mean | SD | CV |
| CiLV-C_CRD/At | 0.848 | 0.985 | 1.020 | 0.864 | 0.921 | 0.963 | 0.933 | 0.068 | 7.280 |
| CiLV-C_CRD/Cs | 0.836 | 0.942 | 1.023 | 0.841 | 0.928 | 0.917 | 0.915 | 0.070 | 7.602 |
| CiLV-C_CRD/On | 1.014 | 1.045 | 0.997 | 1.027 | 0.992 | 1.049 | 1.021 | 0.024 | 2.352 |
| CiLV-C_CRD/Pe | 0.931 | 1.034 | 1.103 | 0.905 | 0.975 | 1.005 | 0.992 | 0.072 | 7.252 |
| CiLV-C_CRD/Phv | 0.782 | 0.909 | 0.938 | 0.808 | 0.896 | 0.844 | 0.863 | 0.061 | 7.101 |
| CiLV-C_SJP/At | 0.841 | 0.901 | 1.035 | 0.907 | 1.005 | 0.918 | 0.934 | 0.072 | 7.709 |
| CiLV-C_SJP/Cs | 0.810 | 0.864 | 1.030 | 0.899 | 1.028 | 0.895 | 0.921 | 0.090 | 9.721 |
| CiLV-C_SJP/On | 0.817 | 0.897 | 1.034 | 0.897 | 0.974 | 0.863 | 0.914 | 0.078 | 8.536 |
| CiLV-C_SJP/Pe | 0.905 | 0.954 | 1.122 | 0.948 | 1.080 | 0.961 | 0.995 | 0.085 | 8.579 |
| CiLV-C_SJP/Phv | 0.772 | 0.825 | 0.944 | 0.845 | 1.007 | 0.815 | 0.868 | 0.089 | 10.244 |
| CiLV-C_Asu/At | 0.846 | 0.968 | 1.027 | 0.890 | 0.929 | 0.968 | 0.938 | 0.064 | 6.856 |
| CiLV-C_Asu/Cs | 0.835 | 0.934 | 1.021 | 0.870 | 0.949 | 0.976 | 0.931 | 0.068 | 7.323 |
| CiLV-C_Asu/On | 0.843 | 0.941 | 1.015 | 0.869 | 0.917 | 0.935 | 0.920 | 0.060 | 6.564 |
| CiLV-C_Asu/Pe | 0.930 | 1.013 | 1.120 | 0.940 | 0.994 | 1.018 | 1.003 | 0.068 | 6.829 |
| CiLV-C_Asu/Phv | 0.780 | 0.901 | 0.958 | 0.832 | 0.899 | 0.899 | 0.878 | 0.062 | 7.114 |
| CiLV-C2_Co/At | 0.829 | 0.911 | 0.949 | 0.880 | 0.932 | 0.951 | 0.908 | 0.047 | 5.196 |
| CiLV-C2_Co/Cs | 0.809 | 0.916 | 0.931 | 0.863 | 0.932 | 0.947 | 0.900 | 0.053 | 5.885 |
| CiLV-C2_Co/On | 0.831 | 0.949 | 0.921 | 0.853 | 0.917 | 0.964 | 0.906 | 0.053 | 5.849 |
| CiLV-C2_Co/Pe | 0.878 | 1.027 | 0.978 | 0.909 | 1.019 | 1.007 | 0.970 | 0.062 | 6.417 |
| CiLV-C2_Co/Phv | 0.772 | 0.835 | 0.880 | 0.808 | 0.857 | 0.862 | 0.836 | 0.040 | 4.800 |
| CiLV-C2_Hw/At | 0.835 | 0.835 | 0.946 | 0.860 | 0.875 | 0.913 | 0.877 | 0.045 | 5.098 |
| CiLV-C2_Hw/Cs | 0.798 | 0.929 | 0.940 | 0.845 | 0.882 | 0.886 | 0.880 | 0.053 | 6.022 |
| CiLV-C2_Hw/On | 0.809 | 0.961 | 0.936 | 0.848 | 0.897 | 0.895 | 0.891 | 0.056 | 6.255 |
| CiLV-C2_Hw/Pe | 0.872 | 1.019 | 1.026 | 0.909 | 0.962 | 0.936 | 0.954 | 0.061 | 6.374 |
| CiLV-C2_Hw/Phv | 0.753 | 0.861 | 0.866 | 0.796 | 0.815 | 0.838 | 0.822 | 0.043 | 5.210 |
| PfGSV_Snp1/At | 0.833 | 0.950 | 1.101 | 0.865 | 0.969 | 0.948 | 0.944 | 0.094 | 9.946 |
| PfGSV_Snp1/Pe | 0.886 | 1.020 | 1.098 | 0.886 | 1.084 | 0.998 | 0.995 | 0.093 | 9.313 |
| PfGSV_Snp1/On | 0.833 | 0.982 | 1.088 | 0.833 | 0.987 | 0.902 | 0.937 | 0.100 | 10.682 |
| PfGSV_Snp1/Cs | 0.831 | 0.983 | 1.088 | 0.836 | 0.985 | 0.925 | 0.941 | 0.099 | 10.487 |
| PfGSV_Snp1/Phv | 0.774 | 0.910 | 1.047 | 0.801 | 0.903 | 0.863 | 0.883 | 0.097 | 10.989 |
| PfGSV_BJL1/At | 0.821 | 0.922 | 1.040 | 0.850 | 0.944 | 0.944 | 0.920 | 0.077 | 8.416 |
| PfGSV_BJL1/Pe | 0.879 | 0.995 | 1.062 | 0.882 | 1.047 | 0.986 | 0.975 | 0.079 | 8.120 |
| PfGSV_BJL1/On | 0.822 | 0.959 | 1.030 | 0.828 | 0.958 | 0.887 | 0.914 | 0.082 | 9.020 |
| PfGSV_BJL1/Cs | 0.821 | 0.950 | 1.061 | 0.845 | 0.962 | 0.919 | 0.926 | 0.087 | 9.375 |
| PfGSV_BJL1/Phv | 0.771 | 0.868 | 1.017 | 0.786 | 0.879 | 0.867 | 0.865 | 0.088 | 10.122 |
| Mean | 0.836 | 0.940 | **1.011** | 0.866 | 0.951 | 0.928 |  |  |  |
| SD | 0.053 | 0.058 | 0.069 | 0.046 | 0.061 | 0.055 |  |  |  |
| CV | 6.314 | 6.210 | 6.784 | 5.365 | 6.370 | 5.955 |  |  |  |

^1^At: *Arabidopsis thaliana*, Cs: *Citrus* x *sinensis*, Pe: *Passiflora edulis*, On: *Oncidium* sp., and Phv: *Phaseolus vulgaris*. ^2^SD: standard deviation, CV: coefficient of variation.

1. Natural host plants.

| **Virus strain/host^1^** | **RCDI/eRCDI ratio** | | | | | | | | |
| --- | --- | --- | --- | --- | --- | --- | --- | --- | --- |
|  | **ORF** | | | | | | **basic statistical data**^2^ | | |
|  | *RdRp* | *p29* | *p15* | *p61* | *p32* | *p24* | Mean | SD | CV |
| CiLV-C_CRD/Cs | 0.836 | 0.942 | 1.023 | 0.841 | 0.928 | 0.917 | 0.915 | 0.070 | 7.602 |
| CiLV-C_CRD/On | 1.014 | 1.045 | 0.997 | 1.027 | 0.992 | 1.049 | 1.021 | 0.024 | 2.352 |
| CiLV-C_CRD/Pe | 0.931 | 1.034 | 1.103 | 0.905 | 0.975 | 1.005 | 0.992 | 0.072 | 7.252 |
| CiLV-C_SJP/Cs | 0.810 | 0.864 | 1.030 | 0.899 | 1.028 | 0.895 | 0.921 | 0.090 | 9.721 |
| CiLV-C_SJP/On | 0.817 | 0.897 | 1.034 | 0.897 | 0.974 | 0.863 | 0.914 | 0.078 | 8.536 |
| CiLV-C_SJP/Pe | 0.905 | 0.954 | 1.122 | 0.948 | 1.080 | 0.961 | 0.995 | 0.085 | 8.579 |
| CiLV-C_Asu/Cs | 0.835 | 0.934 | 1.021 | 0.870 | 0.949 | 0.976 | 0.931 | 0.068 | 7.323 |
| CiLV-C_Asu/On | 0.843 | 0.941 | 1.015 | 0.869 | 0.917 | 0.935 | 0.920 | 0.060 | 6.564 |
| CiLV-C_Asu/Pe | 0.930 | 1.013 | 1.120 | 0.940 | 0.994 | 1.018 | 1.003 | 0.068 | 6.829 |
| CiLV-C2_Co/Cs | 0.809 | 0.916 | 0.931 | 0.863 | 0.932 | 0.947 | 0.900 | 0.053 | 5.885 |
| CiLV-C2_Co/On | 0.831 | 0.949 | 0.921 | 0.853 | 0.917 | 0.964 | 0.906 | 0.053 | 5.849 |
| CiLV-C2_Co/Pe | 0.878 | 1.027 | 0.978 | 0.909 | 1.019 | 1.007 | 0.970 | 0.062 | 6.417 |
| CiLV-C2_Hw/Cs | 0.798 | 0.929 | 0.940 | 0.845 | 0.882 | 0.886 | 0.880 | 0.053 | 6.022 |
| CiLV-C2_Hw/On | 0.809 | 0.961 | 0.936 | 0.848 | 0.897 | 0.895 | 0.891 | 0.056 | 6.255 |
| CiLV-C2_Hw/Pe | 0.872 | 1.019 | 1.026 | 0.909 | 0.962 | 0.936 | 0.954 | 0.061 | 6.374 |
| PfGSV_Snp1/Pe | 0.886 | 1.020 | 1.098 | 0.886 | 1.084 | 0.998 | 0.995 | 0.093 | 9.313 |
| PfGSV_Snp1/On | 0.833 | 0.982 | 1.088 | 0.833 | 0.987 | 0.902 | 0.937 | 0.100 | 10.682 |
| PfGSV_Snp1/Cs | 0.831 | 0.983 | 1.088 | 0.836 | 0.985 | 0.925 | 0.941 | 0.099 | 10.487 |
| PfGSV_BJL1/Pe | 0.879 | 0.995 | 1.062 | 0.882 | 1.047 | 0.986 | 0.975 | 0.079 | 8.120 |
| PfGSV_BJL1/On | 0.822 | 0.959 | 1.030 | 0.828 | 0.958 | 0.887 | 0.914 | 0.082 | 9.020 |
| PfGSV_BJL1/Cs | 0.821 | 0.950 | 1.061 | 0.845 | 0.962 | 0.919 | 0.926 | 0.087 | 9.375 |
| Mean | 0.857 | 0.967 | **1.030** | 0.883 | 0.975 | 0.946 |  |  |  |
| SD | 0.054 | 0.048 | 0.062 | 0.048 | 0.055 | 0.051 |  |  |  |
| CV | 6.253 | 4.958 | 6.068 | 5.419 | 5.648 | 5.395 |  |  |  |

^1^Cs: *Citrus* x *sinensis*, Pe: *Passiflora edulis*, and On: *Oncidium* sp. ^2^SD: standard deviation, CV: coefficient of variation.

1. Experimental host plants.

| **Virus strain/host^1^** | **RCDI/eRCDI ratio** | | | | | | | | |
| --- | --- | --- | --- | --- | --- | --- | --- | --- | --- |
|  | **ORF** | | | | | | **basic statistical data**^2^ | | |
|  | *RdRp* | *p29* | *p15* | *p61* | *p32* | *p24* | Mean | SD | CV |
| CiLV-C_CRD/At | 0.848 | 0.985 | 1.020 | 0.864 | 0.921 | 0.963 | 0.933 | 0.068 | 7.280 |
| CiLV-C_CRD/Phv | 0.782 | 0.909 | 0.938 | 0.808 | 0.896 | 0.844 | 0.863 | 0.061 | 7.101 |
| CiLV-C_SJP/At | 0.841 | 0.901 | 1.035 | 0.907 | 1.005 | 0.918 | 0.934 | 0.072 | 7.709 |
| CiLV-C_SJP/Phv | 0.772 | 0.825 | 0.944 | 0.845 | 1.007 | 0.815 | 0.868 | 0.089 | 10.244 |
| CiLV-C_Asu/At | 0.846 | 0.968 | 1.027 | 0.890 | 0.929 | 0.968 | 0.938 | 0.064 | 6.856 |
| CiLV-C_Asu/Phv | 0.780 | 0.901 | 0.958 | 0.832 | 0.899 | 0.899 | 0.878 | 0.062 | 7.114 |
| CiLV-C2_Co/At | 0.829 | 0.911 | 0.949 | 0.880 | 0.932 | 0.951 | 0.908 | 0.047 | 5.196 |
| CiLV-C2_Co/Phv | 0.772 | 0.835 | 0.880 | 0.808 | 0.857 | 0.862 | 0.836 | 0.040 | 4.800 |
| CiLV-C2_Hw/At | 0.835 | 0.835 | 0.946 | 0.860 | 0.875 | 0.913 | 0.877 | 0.045 | 5.098 |
| CiLV-C2_Hw/Phv | 0.753 | 0.861 | 0.866 | 0.796 | 0.815 | 0.838 | 0.822 | 0.043 | 5.210 |
| PfGSV_Snp1/At | 0.833 | 0.950 | 1.101 | 0.865 | 0.969 | 0.948 | 0.944 | 0.094 | 9.946 |
| PfGSV_Snp1/Phv | 0.774 | 0.910 | 1.047 | 0.801 | 0.903 | 0.863 | 0.883 | 0.097 | 10.989 |
| PfGSV_BJL1/At | 0.821 | 0.922 | 1.040 | 0.850 | 0.944 | 0.944 | 0.920 | 0.077 | 8.416 |
| PfGSV_BJL1/Phv | 0.771 | 0.868 | 1.017 | 0.786 | 0.879 | 0.867 | 0.865 | 0.088 | 10.122 |
| Mean | 0.804 | 0.899 | **0.983** | 0.842 | 0.917 | 0.899 |  |  |  |
| SD | 0.034 | 0.049 | 0.068 | 0.038 | 0.054 | 0.051 |  |  |  |
| CV | 4.284 | 5.503 | 6.867 | 4.505 | 5.865 | 5.672 |  |  |  |

^1^At: *Arabidopsis thaliana*, and Phv: *Phaseolus vulgaris*. ^2^SD: standard deviation, CV: coefficient of variation.

|  |  |  |  |  |  |  |  |  |  |
| --- | --- | --- | --- | --- | --- | --- | --- | --- | --- |
|  |  |  |  |  |  |  |  |  |  |
|  |  |  |  |  |  |  |  |  |  |
|  |  |  |  |  |  |  |  |  |  |
|  |  |  |  |  |  |  |  |  |  |
|  |  |  |  |  |  |  |  |  |  |
|  |  |  |  |  |  |  |  |  |  |
|  |  |  |  |  |  |  |  |  |  |
|  |  |  |  |  |  |  |  |  |  |
|  |  |  |  |  |  |  |  |  |  |
|  |  |  |  |  |  |  |  |  |  |
|  |  |  |  |  |  |  |  |  |  |
|  |  |  |  |  |  |  |  |  |  |
|  |  |  |  |  |  |  |  |  |  |
|  |  |  |  |  |  |  |  |  |  |
|  |  |  |  |  |  |  |  |  |  |
|  |  |  |  |  |  |  |  |  |  |
|  |  |  |  |  |  |  |  |  |  |
|  |  |  |  |  |  |  |  |  |  |
|  |  |  |  |  |  |  |  |  |  |
|  |  |  |  |  |  |  |  |  |  |

**Supplementary Table S7**. Percentage of amino acid sequence identity among cysteine-rich domains in the P15 family of proteins.

|  |  | **1** | **2** | **3** | **4** | **5** | **6** | **7** | **8** | **9** | **10** | **11** | **12** | **13** |
| --- | --- | --- | --- | --- | --- | --- | --- | --- | --- | --- | --- | --- | --- | --- |
| **1** | CiLV-C_Crd01  ABC75823.1 | 100 | 100 | 100 | 98.23 | 24.29 | 22.22 | 22.22 | 23.23 | 22.22 | 19.80 | 19.80 | 22.77 | 22.77 |
| **2** | CiLV-C_SJP01  AKJ79135.1 | 100 | 100 | 100 | 98.23 | 24.29 | 22.22 | 22.22 | 23.23 | 22.22 | 19.80 | 19.80 | 22.77 | 22.77 |
| **3** | CiLV-C_Prb2 | 100 | 100 | 100 | 98.23 | 24.29 | 22.22 | 22.22 | 23.23 | 22.22 | 19.80 | 19.80 | 22.77 | 22.77 |
| **4** | CiLV-C_Ar02  ALF45382.1 | 98.23 | 98.23 | 98.23 | 100 | 24.29 | 22.22 | 22.22 | 23.23 | 22.22 | 19.80 | 19.80 | 22.77 | 22.77 |
| **5** | PfGSV_Cmp1 | 24.49 | 24.49 | 24.49 | 24.49 | 100 | 86.0 | 86.0 | 87.0 | 84.0 | 57.0 | 57.0 | 58.0 | 57.0 |
| **6** | PfGSV_BSB1  QFU28432.1 | 22.22 | 22.22 | 22.22 | 22.22 | 86.0 | 100 | 100 | 87.13 | 84.16 | 63.37 | 63.37 | 64.36 | 63.37 |
| **7** | PfGSV_Snp1  QFU28425.1 | 22.22 | 22.22 | 22.22 | 22.22 | 86.0 | 100 | 100 | 87.13 | 84.16 | 63.37 | 63.37 | 64.36 | 63.37 |
| **8** | PfGSV_BJL1  QFU28439.1 | 23.23 | 23.23 | 23.23 | 23.23 | 87.13 | 87.13 | 87.13 | 100 | 97.03 | 59.41 | 59.41 | 60.40 | 59.41 |
| **9** | PfGSV_Py1 | 22.22 | 22.22 | 22.22 | 22.22 | 84.0 | 84.16 | 84.16 | 97.03 | 100 | 58.42 | 58.42 | 58.42 | 58.42 |
| **10** | CiLV-C2_Co  YP009508072.1 | 19.80 | 19.80 | 19.80 | 19.80 | 57.0 | 63.37 | 63.37 | 59.41 | 58.42 | 100 | 99.03 | 79.61 | 80.58 |
| **11** | CiLV-C2_Co2 | 19.80 | 19.80 | 19.80 | 19.80 | 57.0 | 63.37 | 63.37 | 59.41 | 58.42 | 99.03 | 100 | 79.61 | 80.58 |
| **12** | CiLV-C2_Fla  ATW76025.1 | 22.77 | 22.77 | 22.77 | 22.77 | 58.0 | 64.36 | 64.36 | 60.40 | 58.42 | 79.61 | 79.61 | 100 | 97.09 |
| **13** | CiLV-C2_Hw  AGM16553.1 | 22.77 | 22.77 | 22.77 | 22.77 | 57.0 | 63.37 | 63.37 | 59.41 | 58.42 | 80.58 | 80.58 | 97.09 | 100 |

**Supplementary Table S8**. Summary of the I-TASSER structural predictions for the representative sequences in the P15 family of proteins.

| **P15 sequence** | **C-score** | **estimated TM-score** | **estimated RMSD** | **structural analog**  **(rank 1)** | **RMSD^a^ (Å)** | **IDEN** | **COV** | **Description** |
| --- | --- | --- | --- | --- | --- | --- | --- | --- |
| CiLV-C_Ar02  ALF45382.1 | -2.82 | 0.39±0.13 | 10.8±4.6Å | 5ipx_A | 2.18 | 14.9% | 0.931 | ORF49 human gammaherpesvirus 8 |
| CiLV-C_SJP01  AKJ79135.1 | -3.31 | 0.35±0.12 | 12.0±4.4Å | 3cax_A | 2.56 | 3.1% | 0.977 | Uncharacterized protein PF0695 |
| PfGSV_Cmp1 | -4.04 | 0.28±0.09 | 14.0±3.9Å | 2fkn_B | 4.08 | 4.8% | 0.908 | Urocanate hydratase |
| PfGSV_Snp1  QFU28425.1 | -4.29 | 0.26±0.08 | 14.6±3.7Å | 2b1g_A | 4.21 | 3.4% | 0.893 | inosine monophosphate cyclohydrolase |
| PfGSV_BJL1  QFU28439.1 | -3.52 | 0.33±0.11 | 12.6±4.3Å | 5m98_A | 3.13 | 11.8% | 0.908 | Urate oxidase |
| PfGSV_PY1 | -4.27 | 0.26±0.08 | 14.6±3.7Å | 1vsy_4 | 4.00 | 11.8% | 0.939 | Proteasome component PRE6 |
| CiLV-C2_Co  YP_009508072.1 | -4.37 | 0.26±0.08 | 14.8±3.6Å | 4fio_A | 4.48 | 5.4% | 0.808 | Methenyltetrahydromethanopterin cyclohydrolase |
| CiLV-C2_Co2 | -4.32 | 0.26±0.08 | 14.7±3.6Å | 3s4w_A | 4.28 | 0.8% | 0.923 | Fanconi anemia I |
| CiLV-C2_Fla  ATW76025.1 | -3.90 | 0.29±0.09 | 13.6±4.0Å | 3pzd_A | 3.36 | 8.9% | 0.939 | Myosin X |
| CiLV-C2_Hw  AGM16553.1 | -4.19 | 0.27±0.08 | 14.3±3.8Å | 5yfp_F | 3.84 | 4.9% | 0.815 | Exocyst complex component SEC15 |

C-score: is a confidence score for estimating the quality of predicted models by I-TASSER. It is typically in the range of [-5.2]. where a C-score of higher value signifies a model with high confidence and vice-versa.

TM-score: measures the structural similarity between two structures (Zhang and Skolnick, 2004). TM-score solves the problem of RMSD, which is sensitive to local errors.

RMSD: is the average distance of all residue pairs in two structures.

RMSDa: is the RMSD between residues that are structurally aligned by TM-align.

IDEN: is the percentage sequence identity in the structurally aligned region.

COV: represents the coverage of the alignment by TM-align and is equal to the number of structurally aligned residues divided by the length of the query protein.

**Supplementary Table S9**. A summary of statistics generated by the detection of overlapping genes by the *in silico* method described by Schlub et al. (2018).

| **ORF/virus** | **aa_length** | ***p*val_permute** | ***p*val_synon** | **frameship** | **nstart** | **end** |
| --- | --- | --- | --- | --- | --- | --- |
| *p8*/CiLV-C_CRD | 75 | 0.064 | 0.012232 | plus_1 | 95 | 322 |
| *p8*/CiLV-C_SJP | 75 | 0.071 | 0.019187 | plus_1 | 95 | 322 |
| *p8*/CiLV-C_ASU | 75 | 0.072 | 0.017773 | plus_1 | 95 | 322 |
| *p6*/WhIV8 | 49 | 0.045 | 0.462691 | rev_plus_2 | 45 | 194 |
| *p8*/WHCV1^1^ | 109 | 0.107 | 0.083396 | rev_plus_1 | 146 | 475 |

^1^ Fewer odds than the rest of the detected overlapping genes according to the *p*val_permutation values.
